# Mitochondrial fission, integrity and completion of mitophagy require separable functions of Vps13D in *Drosophila* neurons

**DOI:** 10.1371/journal.pgen.1009731

**Published:** 2021-08-12

**Authors:** Ryan Insolera, Péter Lőrincz, Alec J. Wishnie, Gábor Juhász, Catherine A. Collins

**Affiliations:** 1 Molecular, Cellular and Developmental Biology Department, University of Michigan, Ann Arbor, Michigan, United States of America; 2 Department of Anatomy, Cell and Developmental Biology, Eötvös Loránd University, Budapest, Hungary; 3 Premium Postdoctoral Research Program, Hungarian Academy of Sciences, Budapest, Hungary; 4 Institute of Genetics, Biological Research Centre, Szeged, Hungary; University of Cologne, GERMANY

## Abstract

A healthy population of mitochondria, maintained by proper fission, fusion, and degradation, is critical for the long-term survival and function of neurons. Here, our discovery of mitophagy intermediates in fission-impaired *Drosophila* neurons brings new perspective into the relationship between mitochondrial fission and mitophagy. Neurons lacking either the ataxia disease gene Vps13D or the dynamin related protein Drp1 contain enlarged mitochondria that are engaged with autophagy machinery and also lack matrix components. Reporter assays combined with genetic studies imply that mitophagy both initiates and is completed in Drp1 impaired neurons, but fails to complete in Vps13D impaired neurons, which accumulate compromised mitochondria within stalled mito-phagophores. Our findings imply that in fission-defective neurons, mitophagy becomes induced, and that the lipid channel containing protein Vps13D has separable functions in mitochondrial fission and phagophore elongation.

## Author summary

Neurons are reliant on the maintenance of mitochondrial function to fuel their high-energy demands, and mitochondrial dysfunction is a common pathology associated with neurodegenerative disease. The Vps13 family of proteins are hypothesized to mediate lipid transport between organelles and have been linked to multiple neurological diseases. In *Drosophila* neurons, we delineate separable functions for the essential family member Vps13D in (1) mitochondrial fission, and (2) mitochondrial degradation via mitophagy, which becomes induced in response to fission defects in neurons. Prior studies in *Drosophila* intestinal cells have suggested that Vps13D is required for developmental clearance of mitochondria downstream of its role in mitochondrial fission. In contrast, we find that in neurons, rather than blocking mitophagy, impairments in fission lead to an induction in mitophagy. Vps13D depleted neurons initiate but fail to complete mitophagy, leading to the accumulation of stalled, toxic mitophagy intermediates with compromised integrity. These studies establish a new experimental framework for studying mitophagy in neurons *in vivo*.

## Introduction

Neurons are among the most sensitive cell types to mitochondrial perturbation, and are heavily reliant upon a proper balance of mitochondrial biogenesis and degradation, as well as fission and fusion dynamics [1]. Mutations that disrupt mitochondrial fission and fusion machinery lead to severe neurological dysfunction in humans and animal models [2–4]. Likewise, quality control mechanisms, including the autophagic clearance of damaged mitochondria, known as mitophagy [5], play important protective roles in neurons; impaired mitophagy is thought to contribute to pathology of multiple neurodegenerative diseases [6].

Research in the past decade has uncovered numerous cellular components of mitophagy machinery, primarily through studies that follow toxin-induced damage to the entire mitochondrial population [7–10]. However, neurons strongly require mitochondria, so are unlikely to undergo widespread clearance of mitochondria or survive such harsh insults [11,12]. Instead, neurons are expected to selectively degrade only damaged mitochondria, however molecular tools to study this type of mitophagy in neurons have been limited.

The development of specialized acid-sensitive fluorescent reporters have opened opportunities to monitor mitophagy *in vivo* [13–16], and have thus far been tested in conditions shown to alter *in vitro* toxin-induced mitophagy, with mixed results. Different reporters, mitoQC and mitoKeima, targeted to the outer mitochondrial membrane (OMM) or matrix, respectively [15,17,18], yielded different interpretations of *in vivo* phenotypes. While some of these differences can be attributed to the use of different reporters and model organisms [19], the range of differences in existing studies emphasizes the remaining large gap in our understanding of mitophagy mechanisms in neurons.

Another potential discrepancy between mitophagy in cultured cells compared to neurons *in vivo* is the understood role of mitochondrial fission. Multiple studies [20–22], with some exceptions [23], have indicated that mitochondrial fission is required for the induction of mitophagy in cultured cells subjected to toxins. Conditional knockout of the essential fission protein dynamin-related protein 1 (Drp1) in Purkinje cells of the mouse cerebellum results in the accumulation of autophagy components (ubiquitin, p62, and microtubule-associated proteins 1A/1B light chain 3B (LC3)) on mitochondria [24,25]. These observations suggest that fission via Drp1 is not required for the initiation of mitophagy in neurons, however an understanding of Drp1’s role requires a better understanding of what these autophagy-targeted mitochondria, termed halted mitophagy intermediates [26], represent. One possibility is that Drp1 loss leads to induction of mitophagy, which allows detection of the intermediates because of their frequency. An opposite possibility, which was inferred by these reports, is that Drp1 loss leads to a block in the progression of mitophagy, causing the accumulation of stalled intermediates in the cerebellum. Further *in vivo* studies are needed, with the acknowledgment that mechanisms that support the survival of neurons over the long life time of an animal may diverge considerably from mechanisms sufficient in cultured cells.

Here we have used *Drosophila melanogaster* to investigate the relationship of mitochondrial fission with mitophagy within an *in vivo* nervous system. Our starting point was to understand the neuronal function of the neurodegenerative disease-associated protein Vps13D (Vacuolar Protein Sorting protein 13D). The Vps13 protein family (Vps13A-D) has been characterized as phospholipid transporters that specifically localize to inter-organelle contact sites [27,28]. While a specific cellular function has not yet been established for each of the Vps13 proteins, it is clear that they are all critical for neuronal health, as mutations in all family members are associated with neurological disorders in humans [29–31].

In 2018 our collaborators and others identified *VPS13D* as a cause of familial ataxia [30], developmental movement disorders [30,31] and spastic paraplegia [32]. We found that Vps13D depleted neurons accumulate severely enlarged mitochondria which fail to be trafficked to distal axons [30]. Indeed, loss of Vps13D function in many different cell types leads to severely enlarged mitochondria, including *Drosophila* intestinal cells, HeLa cells [33,34], and cultured fibroblasts from human ataxia patients containing point mutations in the *VPS13D* gene [30]. These findings suggest a conserved role for Vps13D in mitochondrial dynamics.

However, in neurons we noticed additional phenotypes associated with loss of Vps13D. Here we report the accumulation of objects that appear to be stalled intermediates which have initiated but fail to complete mitophagy. These intermediates lack matrix components, hence evade detection by traditional means of mitochondrially targeted reporters. This led us to question whether these objects also appear in other genetic conditions. Indeed, we found similar objects in neurons mutant or depleted for Drp1. Through our study of mitophagy in the fission-deficient conditions of Drp1 or Vps13D depletion, and found that: (1) neurons deficient in their capacity for mitochondrial fission can still undergo functional mitophagy; (2) neurons require Vps13D for a separable function in phagophore elongation, in addition to mitochondrial fission; and (3) mitophagy within neurons is robustly induced following impairments in mitochondrial fission. These studies establish a new experimental framework for studying the induction and completion of mitophagy in neurons *in vivo*.

## Results

### Neurons depleted for Vps13D contain mitophagy intermediates that have lost matrix components

**Fig 1:**
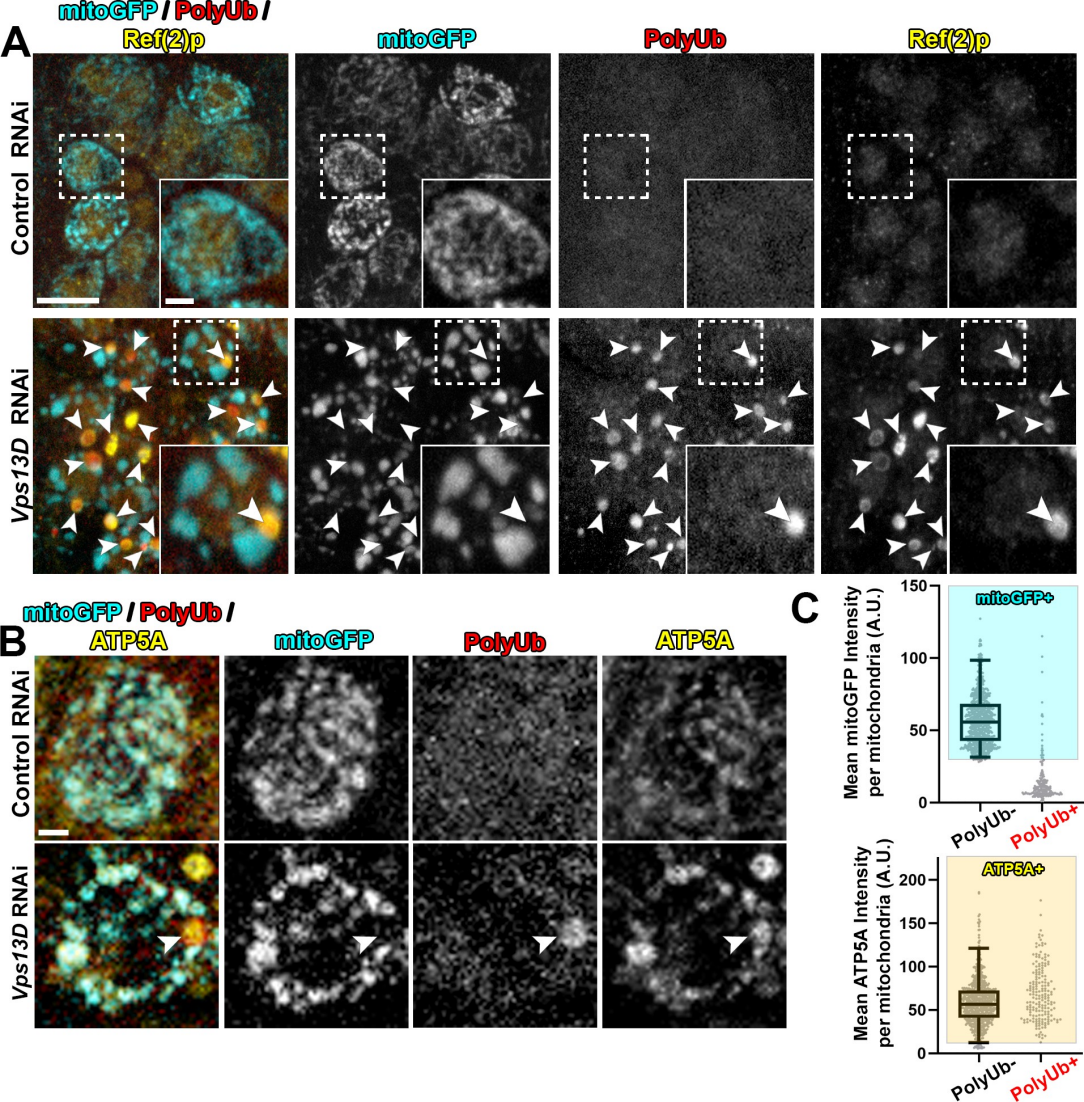
Neurons depleted for Vps13D contain mitophagy intermediates lacking matrix. Representative images of motoneurons in the larval VNC which co-express UAS-mitoGFP (cyan) with UAS-luciferase RNAi (control) (BL# 31603) versus UAS-Vps13D RNAi (BL# 38320), via the D42-Gal4 driver. **A**) VNC tissue was stained for polyubiquitin (PolyUb, FK1, red) and Ref(2)p (p62 homolog, yellow). Dashed box outlines a single Gal4-expressing neuronal cell body that is shown in high magnification in the inset in the bottom right corner. Arrowheads indicate examples of PolyUb+ objects (PolyUb+/Ref(2)p+/mitoGFP-). Vps13D RNAi expressing neurons contained on average 1.88 PolyUb+ objects per cell body (n = 239 neurons from 5 ventral nerve cords (VNCs)), while such objects were never observed in neurons expressing control RNAi. 98.8% of PolyUb+ objects were Ref(2)p+, n = 485 PolyUb+ objects from 5 larval VNCs. Scale bars = 10μm, 2μm. **B)** Co-staining for mitoGFP (cyan), polyubiquitin (PolyUb, red) and endogenous IMM protein ATP5A (yellow). The arrowhead highlights an example PolyUb+ object, which contains ATP5A, but lacks mitoGFP. Scale bar = 2μm. **C)** Distribution of mean intensities for mitoGFP (top) and ATP5A (bottom) from mitochondria in motoneurons. The right column shows objects selected based on positivity for PolyUb. The left column shows the population of conventional (PolyUb-) mitochondria (selected by similar criteria). Based on the criteria of >2.5% of the intensity distribution of the PolyUb- mitochondria population (shaded boxes), PolyUb+ objects were classified as + for mitoGFP and/or ATP5A if they were within the shaded box. n = 809 mitochondria and 184 PolyUb+ objects from 5 larval VNCs.

Consistent with our previous work [30], and previous observations in intestinal cells [33], RNAi depletion of Vps13D in larval motoneurons led to severely enlarged neuronal mitochondria, as visualized by Gal4-driven expression of matrix-targeted GFP (mitoGFP) [35,36] (**Fig 1A**). We also noticed large, round polyubiquitin (PolyUb) positive objects that were of similar size to the enlarged mitochondria, but lacked mitoGFP (**Fig 1A**). The PolyUb+ objects were also strongly positive for Ref(2)p, the Drosophila homolog of mammalian p62 (**Fig 1A**).

We hypothesized that the PolyUb+/Ref(2)p+ objects are comprised of cellular substrates that are incompletely degraded in conditions of Vps13D depletion. To our surprise, we observed that PolyUb+ objects stained positive for the endogenous inner-mitochondrial membrane (IMM) protein ATP5A (**Fig 1B**). Of these enlarged PolyUb+ objects in the neuronal cell bodies, only 9.8% had mitoGFP intensities that fell within the range of the traditional neuronal mitochondria in this condition, which were PolyUb-, but contained clear mitoGFP and ATP5A staining (**Fig 1C**). However, all (100%) of these PolyUb+ objects had ATP5A intensities within the range of these traditional neuronal mitochondria. We observed similar Ref(2)p+/ATP5A+ objects in neurons of *Vps13D* mutant larvae (**S1 Fig**), which died in early larval stages [30,33].

**Fig 2:**
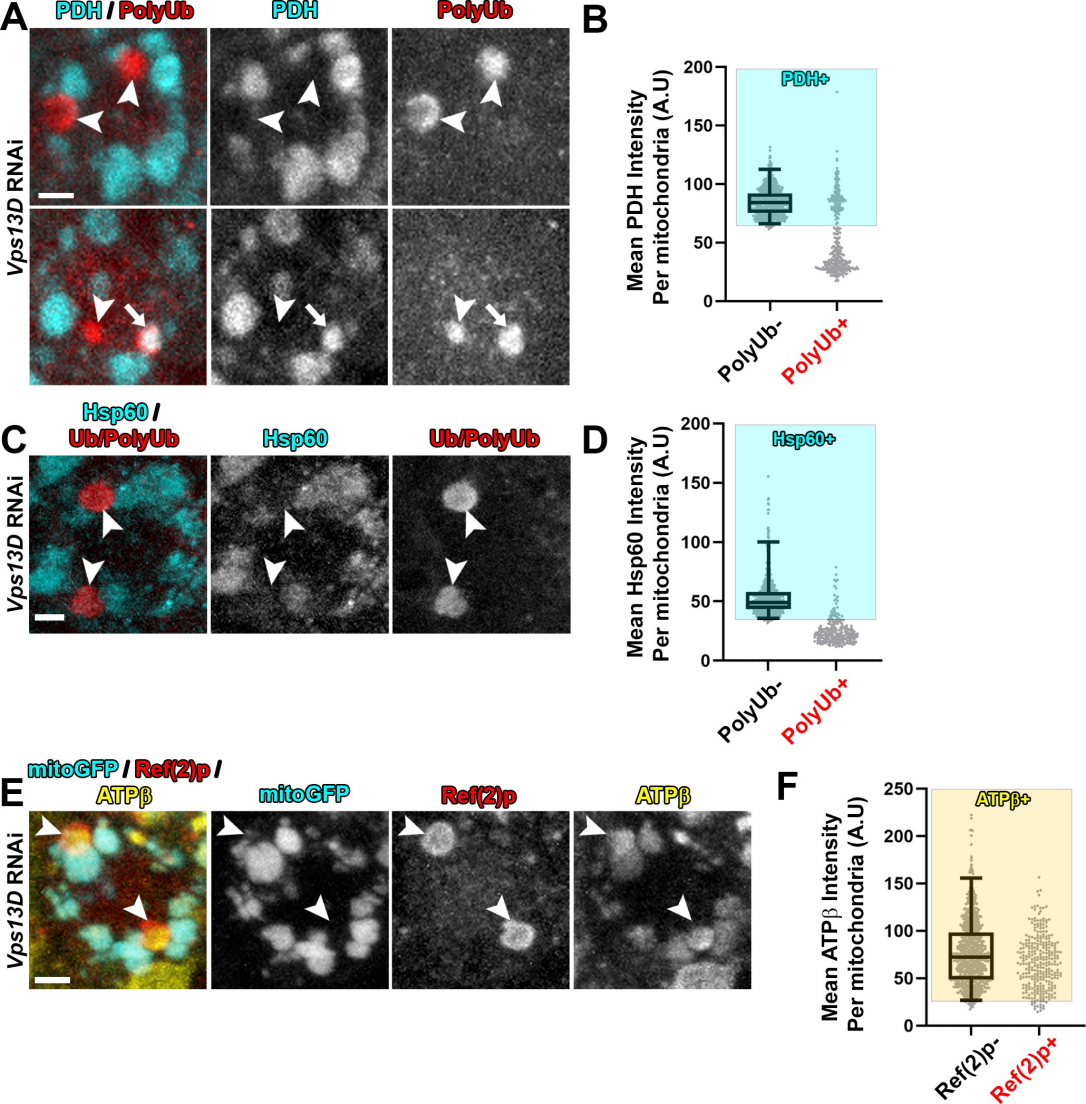
Mitophagy intermediates in Vps13D depleted neurons lack endogenous soluble matrix proteins, but contain endogenous IMM protein. Representative images of single motoneuron cell bodies which express *Vps13D* RNAi via the D42-Gal4 driver. **A,C)** VNC tissue was stained for stained for endogenous mitochondrial matrix proteins pyruvate dehydrogenase E1ɑ (PDH) (A) or Hsp60 (C) (cyan) and PolyUb or Ub/PolyUb (red). Arrowheads highlight examples of PolyUb+ objects lacking matrix staining, while the arrow highlights an example of a PolyUb+ object that contains PDH. Scale bars = 2μm. **B,D)** Distribution of mean intensities for PDH (B) and Hsp60 (D) from mitochondria in motoneurons. The right columns show objects selected based on positivity for PolyUb (n = 351 PolyUb+ objects from 5 larval VNCs for (B) and n = 227 PolyUb+ objects from 5 larval VNCs in (D)). The left columns show the population of conventional (PolyUb-) mitochondria (n = 789 mitochondria from 5 larval VNCs for (B); n = 744 mitochondria from 5 larval VNCs for (D)). Based on the criteria of intensity greater than 2.5% of conventional mitochondria (shading), 33.6% of the PolyUb+ objects contain PDH, while 13.7% contain Hsp60. **E)** VNC tissue was stained for endogenous mitochondrial IMM protein ATPβ (yellow) and Ref(2)p (red). Arrowheads highlight examples of Ref(2)p+ objects that lack mitoGFP but contain ATPβ. Scale bar = 2μm. **F)** Distribution of mean intensities for ATPβ from mitochondria in motoneurons. The right column shows objects selected based on positivity for PolyUb (n = 296 PolyUb+ objects from 5 larval VNCs). The left column shows the population of conventional (PolyUb-) mitochondria (PolyUb-) (n = 883 mitochondria from 5 larval VNCs). Based on the criteria of intensity greater than 2.5% of conventional mitochondria (shading), 94.3% of the PolyUb+ objects contain ATPβ.

Since the mitoGFP marker is derived from an exogenously expressed transgene, we probed for additional mitochondrial components in Vps13D depleted neurons. Immunostaining for the endogenous matrix enzyme pyruvate dehydrogenase E1ɑ (PDH) (**Fig 2A and 2B**) and matrix localized chaperone heat shock protein 60 (Hsp60) (**Fig 2C and 2D**) indicate the absence of these matrix proteins in the majority of PolyUb+/Ref(2)p+ objects (33.3% and 13.7% of the PolyUb+/Ref(2)p+ objects contain PDH or Hsp60, respectively). Consistent with immunostaining of the endogenous IMM protein ATP5A (**Fig 1B**), immunostaining for ATPβ corroborated the presence of IMM proteins in these intermediates (**Fig 2E and 2F**) (94.3% of the PolyUb+ objects contained ATPβ). In addition, exogenous expression of an epitope tagged version of the full-length matrix enzyme isocitrate dehydrogenase 3β (UAS-Idh3b-HA) [37] labels mitochondria, but failed to label the PolyUb+ objects (**S2 Fig**). From these observations we infer that the PolyUb+/Ref(2)p+/ATP5A+/mitoGFP- objects are derived from mitochondria, but the composition of their mitochondrial matrix is aberrant, showing a strong reduction in the levels of soluble matrix proteins.

**Fig 3:**
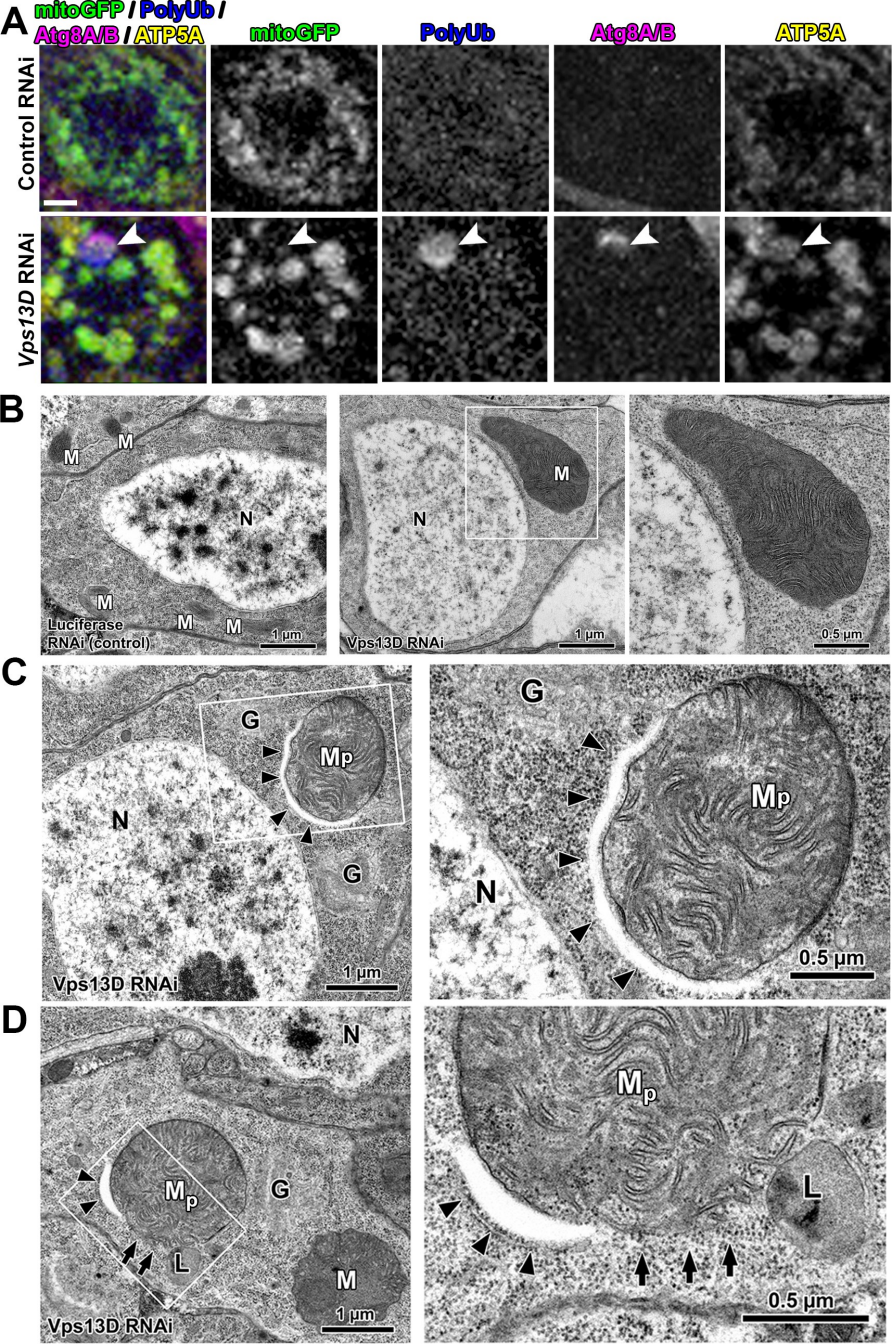
Mitophagy intermediates in Vps13D depleted neurons engage with a phagophore. **A)** Representative images of individual larval motoneurons which co-express mitochondrial marker mitoGFP (green) and the indicated RNAi via the D42-Gal4 driver, stained for polyubiquitin (PolyUb, blue), endogenous Atg8A/B (magenta), and endogenous IMM protein ATP5A (yellow). Arrowhead highlights an example of a mitophagy intermediate engaged with a phagophore but lacking mitochondrial matrix protein marker (PolyUb+/ATP5A+/Atg8+/mitoGFP-). 67.7% of the PolyUb+/ ATP5A+ objects in the neurons lacking Vps13D showed colocalization with Atg8A/B. (n = 425 PolyUb+/ATP5A+ objects from 5 larval VNCs). Scale bar = 2μm **B)** Representative electron micrographs of larval neurons which express RNAi targeting either luciferase (control) or Vps13D, using the pan-neuronal driver Elav-Gal4. Severely enlarged mitochondria (M) in *Vps13D* RNAi condition (right) are readily apparent, in comparison to Control RNAi condition (left). Magnified view of enlarged mitochondria (white box) is shown in the far right panel. Other abbreviations: Golgi (G) and Nucleus (N). **C, D)** Representative electron micrographs showing two examples of phagophore-associated mitochondria (M_p_) found within neurons in the VNC of *Vps13D* RNAi expressing larvae. Panels on the right show a magnified view of the boxed region. Arrowheads indicate the phagophore. In **D**) arrows indicate locations lacking mitochondrial membrane, where mitochondrial content appears to mix with the surrounding cytoplasm. Other abbreviations: Mitochondria (M), Golgi (G), and Lysosome (L). Scale bars indicated in images. Additional example images are provided in **S3 Fig**.

The presence of the autophagy receptor Ref(2)p suggested that the PolyUb+/Ref(2)p+/ATP5A+/mitoGFP- objects may be in the process of mitophagy. Indeed, 67.7% of the PolyUb+/ ATP5A+ objects showed colocalization with the core phagophore component Atg8, stained by antibodies that recognize endogenous Atg8A/B [38] (**Fig 3A**). Similar colocalization of ATP5A and Atg8A/B was observed in *Vps13D* mutant neurons (**S1 Fig**), while no comparable structures were observed in control animals.

We then looked for these phagophore-associated objects by electron microscopy (EM). Micrographs of neuronal cell bodies in the VNC expressing Vps13D RNAi compared to control RNAi revealed strikingly enlarged mitochondria which contain densely packed cristae (**Fig 3B**). Some enlarged mitochondria were associated with a non-electron-dense double membrane consistent with a phagophore (designated as M_p_ in **Figs 3C and S3**). Mitochondria engaged with a phagophore were consistently more disorganized, and less electron dense than neighboring mitochondria in the same micrograph (**S3 Fig**), but contained both IMM and outer-mitochondrial membrane (OMM). Strikingly, in some instances the phagophore-associated mitochondria showed membrane rupture and loss of barrier between the OMM, IMM, and the cytoplasm (**Figs 3D and S3**). While we cannot rule out a fixation artifact, the potential rupture could provide an attractive explanation for the absence of staining for matrix markers.

We never observed mitochondria fully enveloped in what would resemble a completed autophagosome, or localized in lysosomes in micrographs. Three-dimensional rendering of confocal images of Atg8A/B staining identified partially encapsulated mitochondria, however fully encapsulated PolyUb+ mitochondria were never detected in neurons lacking Vps13D (**S4 Fig**). Because these mitochondrial objects are engaged with a phagophore, we hypothesize that the PolyUb+/Ref(2)p+/ATP5A+/Atg8+/mitoGFP- objects are stalled intermediates in mitophagy. Going forward, we refer to these objects as mitophagy intermediates lacking matrix.

### Similar mitophagy intermediates lacking matrix accumulate in neurons disrupted for Drp1

**Fig 4:**
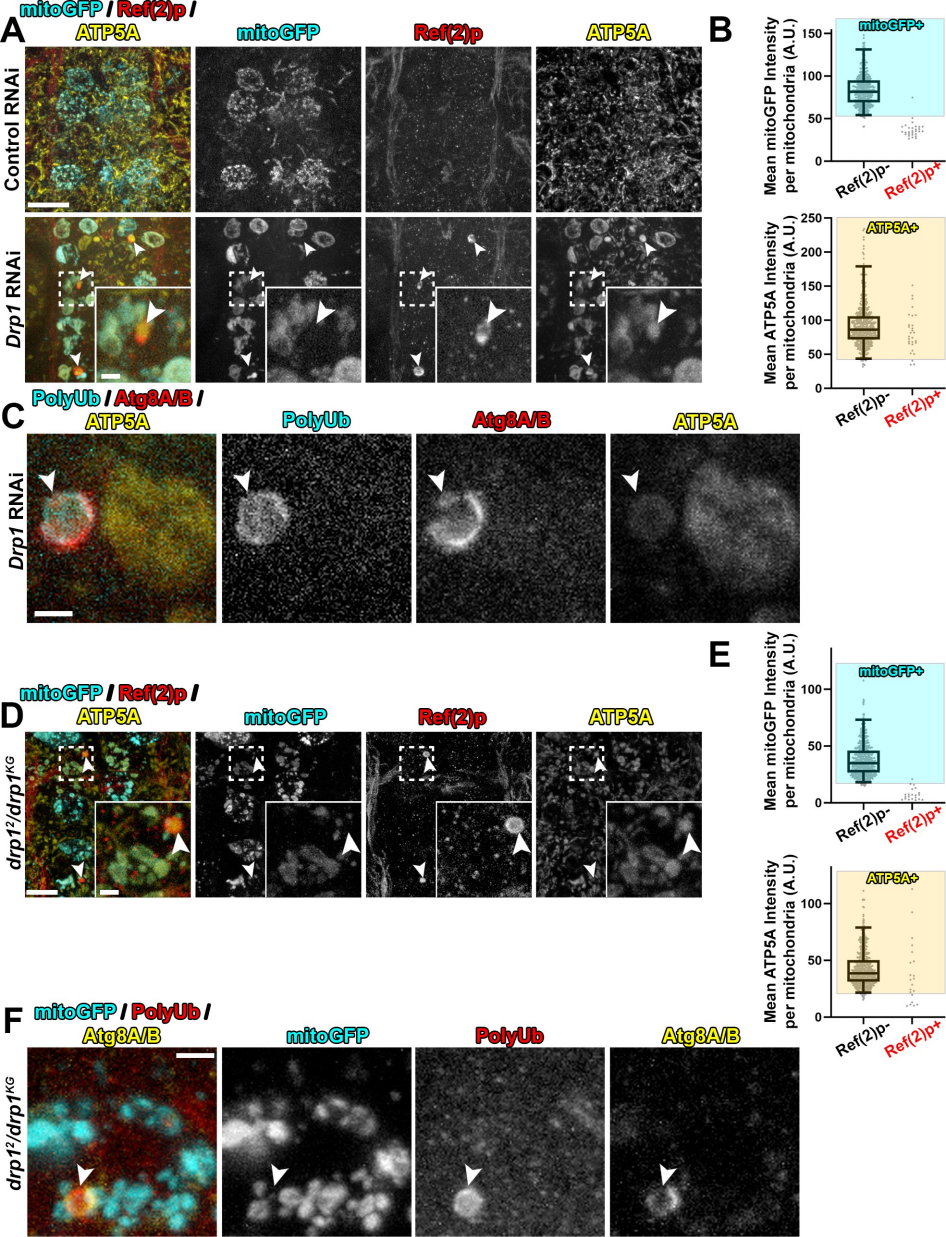
Neurons depleted for Drp1 contain mitophagy intermediates that lack matrix proteins, similar to Vps13D depleted neurons. **A)** Representative images of motoneurons in the larval VNC which co-express the mitochondrial marker mitoGFP (cyan) and RNAi targeting luciferase (control) versus Drp1 (BL# 67160), via the D42-Gal4 driver. Tissue was stained for Ref(2)p (red) and ATP5A (yellow). Dashed box outlines a single Gal4-expressing neuronal cell body that is shown in high magnification via the inset in the bottom right corner. Arrowheads highlight examples of mitophagy intermediates (Ref(2)p+/ATP5A+/mitoGFP-). Scale bars = 10μm, 2μm. **B)** Distribution of intensities for mitoGFP (top) and ATP5A (bottom) in conventional mitochondria (ATP5A+/mitoGFP+ and Ref(2)p-), compared to the Ref(2)p+ objects. Based on the criteria of intensity greater than 2.5% of conventional mitochondria (shading), 3.4% of Ref(2)p+ objects contained mitoGFP, while 89.7% contained ATP5A. n = 528 mitochondria and 29 PolyUb+ from 8 larval VNCs. **C)** Representative image from a *Drp1*-RNAi expressing neuron which contains two enlarged ATP5A+ mitochondria (yellow). One, highlighted by the arrowhead, co-localizes with PolyUb (cyan), and endogenous Atg8A/B (red). 84.2% of PolyUb+/ATP5A+ objects co-localize with Atg8A/B (n = 38 PolyUb+ mitochondria from 15 larval VNCs). Scale bar = 2μm. **D)** Representative image of motoneurons in the larval VNC of *drp1*^*2*^*/drp1*^*KG*^ mutant larvae, with UAS-mitoGFP (cyan) expressed via the D42-Gal4 driver. Dashed box outlines a single Gal4-expressing neuronal cell body that is shown in high magnification in the inset in the bottom right corner. Arrowheads highlight examples of mitophagy intermediates that are positive for Ref(2)p (red) and ATP5A (yellow), but lack mitoGFP (cyan). Scale bars = 10μm, 2μm. **E)** Graph showing the distribution of intensities for mitoGFP (top) and ATP5A (bottom) in conventional mitochondria (ATP5A+/mitoGFP+ and Ref(2)p-), compared to the Ref(2)p+ objects (n = 648 mitochondria and 29 Ref2(p)+ objects from 7 larval VNCs). Based on the criteria of intensity greater than 2.5% of conventional mitochondria (shading), 4.5% of Ref(2)p+ objects contain mitoGFP and 68.2% of Ref(2)p+ objects contain ATP5A. **F)** Representative image of a mitophagy intermediate (arrowhead) in the VNC of a *drp1*^*2*^*/drp1*^*KG*^ mutant larva, with UAS-mitoGFP (cyan) expressed via the D42-Gal4 driver. The highlighted intermediate in this motoneuron stains for PolyUb (red) and endogenous Atg8A/B (yellow), but lacks the matrix marker mitoGFP. 74.4% of PolyUb+/mitoGFP- objects co-localized with Atg8A/B (n = 43 mitophagy intermediates lacking matrix from 8 *drp1* mutant VNCs). Scale bar = 2μm.

Mitophagy intermediates lacking matrix were not previously observed In *Drosophila* intestinal cells depleted for Vps13D [33]. We then wondered whether these intermediates reflect a neuron-specific consequence of impaired mitochondrial fission. We first utilized Gal4-driven motoneuron expression (D42-Gal4) of *Drp1* RNAi [39], simultaneous with expression of mitoGFP in motoneurons. Knockdown of Drp1 resulted in extreme enlargement of mitochondria, to a greater degree than loss of Vps13D (**S5 Fig**), and also led to the presence of Ref(2)p+/ATP5A+/mitoGFP- mitophagy intermediates (**Fig 4A and 4B)**. Further, these mitophagy intermediates are associated with a phagophore, as shown by the colocalization of ATP5A+/PolyUb+ mitochondria with Atg8A/B (**Fig 4C**) 84.2% of the time.

To verify that the mitophagy intermediates are linked to loss of Drp1 function, we examined independent genetic methods to impair Drp1. Based on the morphological enlargement of mitochondria, knockdown of *Drp1* via RNAi is strong, as the phenotype most closely compared to the loss-of-function *drp1*^*KG*^*/Df* mutant condition *(Df = deficiency)* [39,40] **(S5 Fig).** Regardless of the allelic combination, we observed the presence of Ref(2)p+/ATP5A+/mitoGFP- mitophagy intermediates in all *drp1* mutants **(Figs 4D, 4E and** S5**),** with a frequency that correlated with the severity of the mitochondrial enlargement phenotype. Consistent with other control conditions (Control RNAi and *Vps13D* heterozygous animals), these objects were not observed in *drp1* heterozygous animals. Similar to *Vps13D* RNAi, and *Drp1* RNAi conditions, the PolyUb+/mitoGFP- intermediates in *drp1* mutants engage with a phagophore, as they stain positive for Atg8A/B (**Fig 4F**) 74.4% of the time. Overall, these results demonstrate that neurons with either disrupted Vps13D or Drp1 contain mitophagy intermediates that uniquely lack mitochondrial matrix proteins, and are engaged with a phagophore.

### Completion of mitophagy differs between the fission-deficient conditions of Vps13D and Drp1 loss

The presence of mitophagy intermediates could reflect a blockage in mitophagy completion, which was suggested to occur in the cerebellum of *Drp1* knockout mice [24]. To estimate successful trafficking to the lysosome, we turned to fluorescent reporters that contain dual acid-labile (GFP) and acid-stable (mCherry) tags. We chose not to test the mitophagy reporter mitoKiema [18] because mitoGFP, which utilizes the same mitochondrial targeting sequence to localize the acid-sensitive fluorescent protein Keima to the matrix, was largely absent from stalled mitophagy intermediates in Vps13D and Drp1 depleted neurons (**Figs 1B and 4A**). In addition, for unknown reasons, the OMM targeted mitoQC reporter [13,15,41] did not consistently localize to mitophagy intermediates in our experimental conditions **(S6 Fig)**; we did not further optimize the use of this reporter.

We instead turned to a previously characterized mCherry-GFP-Atg8A reporter for autophagy [42–44]. This reporter showed co-localization with the Ref(2)p+ mitochondria in Vps13D and Drp1 depleted neurons (**S7 Fig**), consistent with the endogenous Atg8A/B localization (**Fig 3A**). Dual GFP/mCherry-containing phagophores were only detectable in a small subpopulation of Vps13D depleted neurons in live VNC preparations (**S7 Fig**), likely due to lower fluorescence intensity in active phagophores compared to accumulated signal in lysosomes [45]. However, bright mCherry-only signal, indicating the accumulation of reporter delivered to the acidic lysosome compartment, were detectable in the majority of neurons in all conditions in live preparations. The mCherry-only signal was increased in larval motoneurons overexpressing Atg1, which is expected to induce autophagy [45] (**S8 Fig**). Conversely, knockdown of the essential autophagy component Atg5 resulted in less overall mCherry-only signal in neurons containing mCherry-only puncta (38.5% compared to Control RNAi), with a high proportion of neurons lacking mCherry-only signal altogether (47.8%, 34 of 71 neurons) (**S8 Fig**). These results indicate that the total levels of mCherry-only signal in larval motoneurons expressing the mCherry-GFP-Atg8A reporter can represent an estimation of delivery of autophagic substrate to the lysosome.

**Fig 5:**
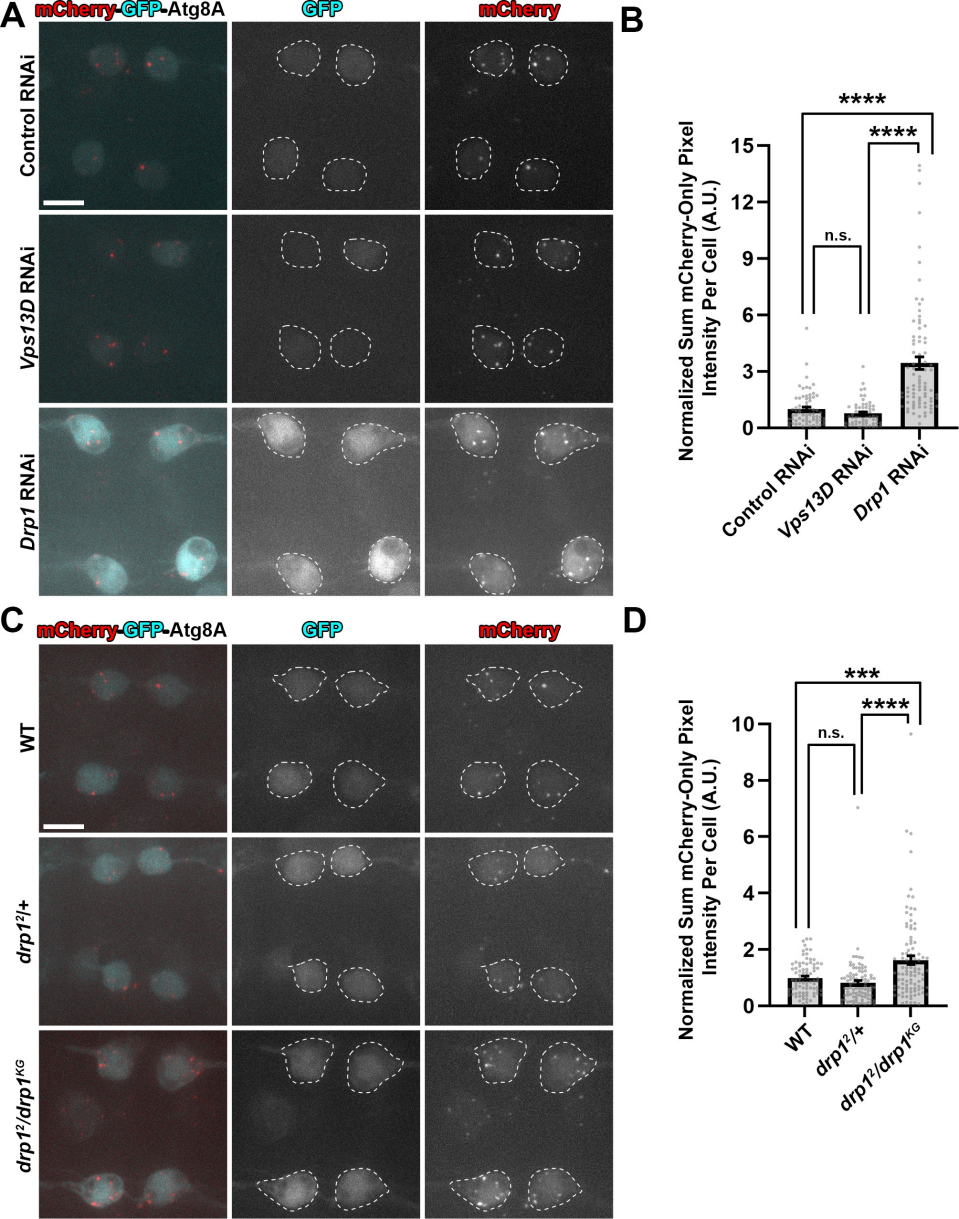
Reporter data imply that Vps13D, but not Drp1, is required for mitophagy completion. **A)** Representative images of live larval motoneurons expressing UAS-mCherry-GFP-Atg8A, expressed via the D42-Gal4, simultaneous with expression of UAS-RNAi lines targeting luciferase (control) (BL# 31603), *Vps13D* (BL# 38320), or *Drp1* (BL# 67160). White dashed lines indicate the outlines of individual cell bodies. Scale bar = 10μm. **B)** Quantification of the sum pixel intensity of the mCherry-only signal per neuronal cell body (normalized to Control RNAi). Each point represents a single neuronal cell body, bars represent the mean ± SEM (Control RNAi n = 69 cell bodies; *Vps13D* RNAi n = 59 cell bodies; and *Drp1* RNAi n = 82 cell bodies from 6 larval VNCs each). **** represents p value <0.0001. n.s. (not significant) represents p value >0.5 (p = 0.09). **C)** Representative images of live larval motoneurons expressing UAS-mCherry-GFP-Atg8A, expressed via the D42-Gal4 driver, in the indicated genetic backgrounds. White dashed lines indicate the outlines of individual cell bodies. Scale bar = 10μm. **D)** Quantification of the sum pixel intensity of the mCherry-only signal per neuronal cell body (normalized to WT). Each point represents a single neuronal cell body, bars represent the mean ± SEM (WT n = 84 cell bodies; *drp1*^*2*^*/*+ n = 93 cell bodies; and *drp1*^*2*^*/drp1*^*KG*^ n = 96 cell bodies from 7 larval VNCs each). *** represents p value <0.001. **** represents p value <0.0001. n.s. (not significant) represents p value >0.5 (p = 0.09).

An experimental condition that has been rigorously demonstrated to induce mitophagy in the *Drosophila* larval nervous system has not been previously established. Based on our observations of mitophagy intermediates in the fission-deficient conditions we tested above, we expected to observe an increased delivery of reporter to lysosomes in both conditions due to elevated mitophagy. However, instead we observed strikingly different results between Vps13D and Drp1 depleted neurons. Drp1 depletion led to a strong induction of mCherry-only mCherry-GFP-Atg8A puncta (magenta in merged image), however this induction was not observed in Vps13D depleted neurons (**Fig 5A and 5B**). Because both Vps13D and Drp1 impaired conditions revealed mitophagy intermediates associated with a phagophore, we interpret that mitophagy was induced in both conditions, however these conditions may differ in their ability to complete clearance via the lysosome.

A potential confound to this interpretation was an unexplained observation that the mCherry-GFP-Atg8A reporter was detected at significantly higher levels in neurons expressing *Drp1* RNAi. We therefore repeated the experiment in *drp1* mutants, which did not show elevated expression levels of the reporter. We found that *drp1* mutant neurons also showed an increased number of mCherry-only puncta compared to WT and heterozygous animals (**Fig 5C and 5D**), and this increase was suppressed in the condition of Atg5 knockdown (**S8 Fig**). Therefore the mCherry-only puncta reflect an increase in autophagic/mitophagic flux in *drp1* mutant conditions (and not simply an increase in reporter expression levels).

The observation that Vps13D depleted neurons still show the presence of mCherry-GFP-Atg8A puncta that fluoresces only in the mCherry channel suggests that at least some degree of macroautophagy still occurs in this condition. This is consistent with the observation that starvation-induced autophagy can still occur in *Vps13D* mutant fat body cells [33]. However, in contrast to Drp1-impaired conditions, we failed to see an increase in mCherry-only puncta, even though the presence of intermediates indicates that mitophagy is initiated in both Vps13D and Drp1-impaired conditions. We infer that Vps13D function may not be essential for macroautophagy but is specifically required for completion of mitophagy, a form of selective autophagy.

### Genetic validation of incomplete mitophagy in Vps13D depleted neurons compared to Drp1 depleted neurons

An independent method to assess whether mitophagy is completed in Vps13D or Drp1 depleted genotypes is to test whether conditions that block autophagy lead to a further accumulation of mitophagy intermediates. To block autophagy in neurons *in vivo* we used a null mutation in the core autophagy component *Atg5 [46]*.

**Fig 6:**
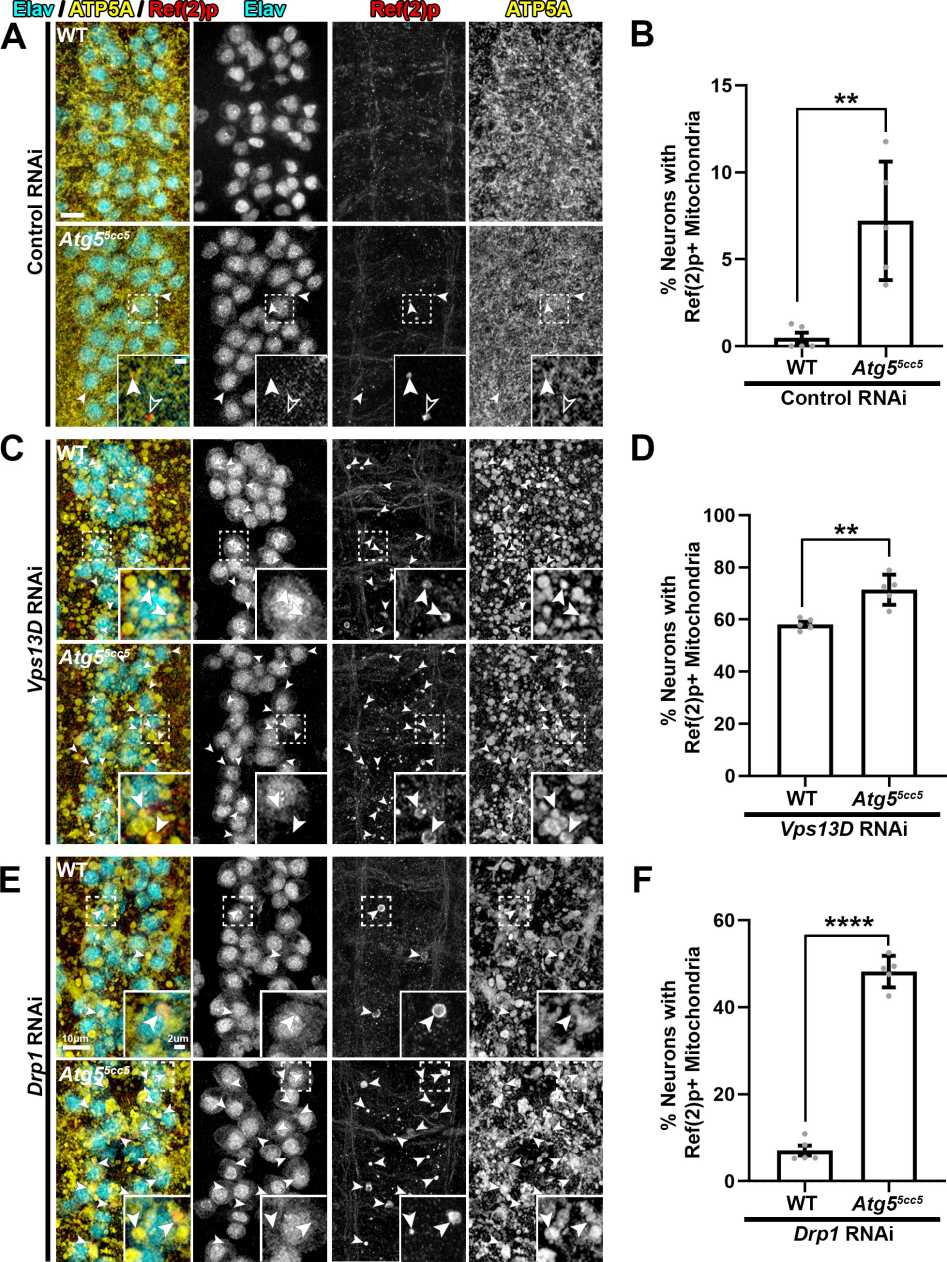
The accumulation of mitophagy intermediates following autophagy blockade implies that Vps13D, but not Drp1, is required for mitophagy completion. **A,C,E)** Representative images of dorsal midline motoneurons in larval VNCs from a wildtype background (*w*^*1118*^) (top) or *Atg5* null (*Atg5*^*5cc5*^) background (bottom) expressing (A) UAS-luciferase (Control) RNAi, (C) UAS-*Vps13D* RNAi, or (E) UAS-*Drp1* RNAi. Tissue was stained for neuron-specific transcription factor Elav (cyan), autophagy receptor protein Ref(2)p (red), and mitochondrial IMM protein ATP5A (yellow). The inset in the bottom right corner shows a high magnification image of a single confocal plane from a neuronal cell body (dashed box). Arrowheads highlight mitophagy intermediates (Ref(2)p+/ATP5A+). In (A), an additional population of Ref2(p)+ puncta that lacked ATP5A (open arrowheads) were detected in *Atg5* null mutants expressing control RNAi. Scale bars = 10μm, 2μm. **B,D,F)** Quantification of the percentage of dorsal midline motoneurons containing a mitophagy intermediate (Ref(2)p+ mitochondria). Grey points represent the % of neurons from one animal (n = 5 animals for each condition). Bars represent mean ± SEM. ** p<0.01; **** p<0.0001.

We first carried out further analysis of mitochondria and potential mitophagy intermediates in motoneurons of *Atg5* null larvae. While mitochondria in *Atg5* null neurons were slightly enlarged in morphology compared to control animals **(S9 Fig)**, the overall complex network of mitochondria remained intact, and the strongly enlarged and isolated mitochondria (present in Vps13D and Drp1 depleted neurons) were not observed. We observed Ref(2)p+ puncta in *Atg5* null neurons, some of which fully overlapped with ATP5A which were scored as mitophagy intermediates (closed arrowhead, compared to open arrowhead). Ref(2)p+/ATP5A+ species were observed in a relatively low percentage of neurons (7.2%) (**Fig 6A and 6B**).

In *Atg5* null neurons expressing *Vps13D* RNAi, the accumulation of phagophore marker Atg8A/B on PolyUb+ mitochondria was abolished (**S9 Fig**), confirming both the effectiveness of *Atg5* deletion in blocking autophagy and the dependence of Atg8A/B staining on autophagy induction. Importantly, in these conditions, mitochondria were still co-localized with PolyUb, suggesting that they form independently to impairments in autophagy machinery.

**Fig 7:**
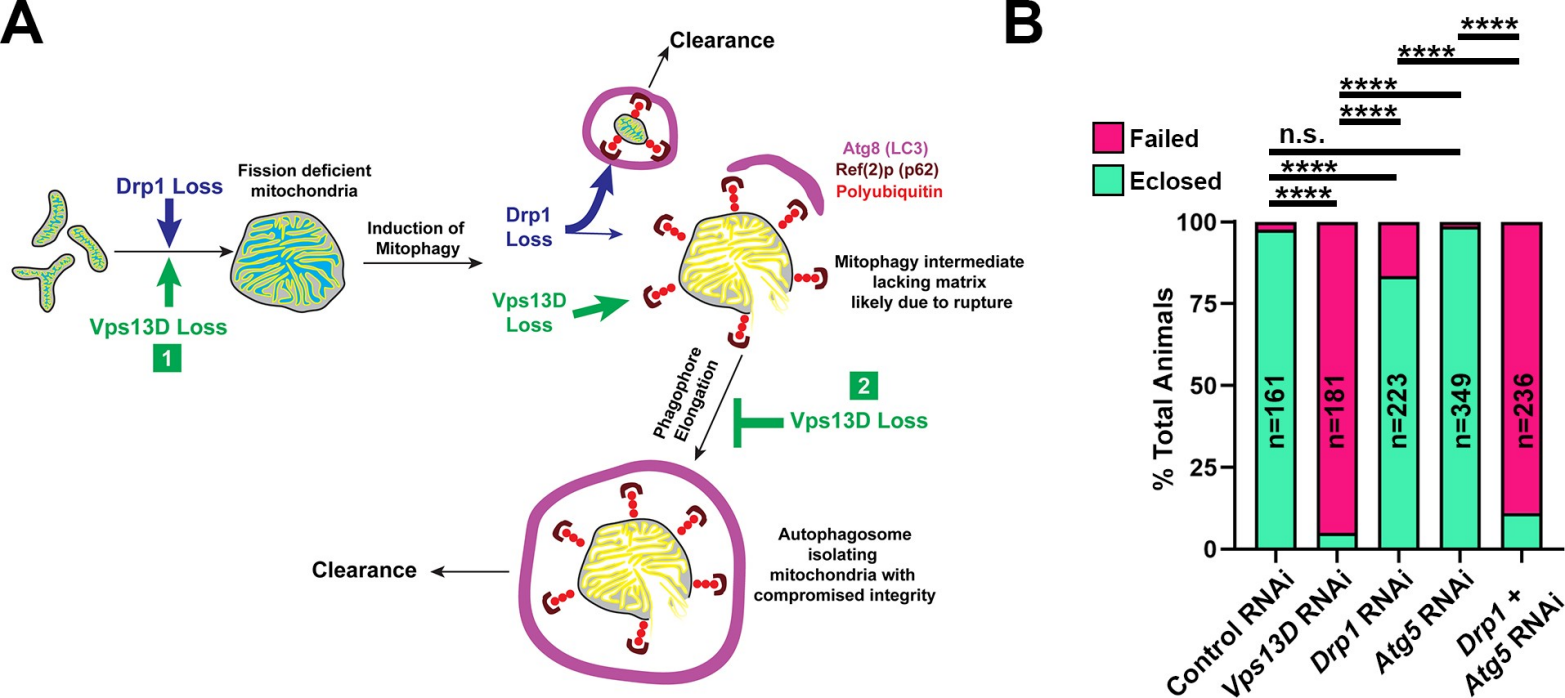
Two-hit model for lethality in Vps13D depleted neurons. **A)** Two-hit model for mitophagy intermediates lacking matrix (cyan), but containing IMM (yellow) that are present in Vps13D depleted neurons. (1) loss of Vps13D and loss of Drp1 lead to similar defects in mitochondrial fission and an induction of mitophagy. In Drp1 depleted neurons (blue arrows and text), mitophagy clears the majority of smaller, matrix-containing mitochondria, while a subpopulation of larger mitophagy intermediates lacking matrix exist that appear stalled. In Vps13D depleted neurons (green arrows and text), a second role for Vps13D is revealed for (2) elongating the phagophore around damaged mitochondria. Therefore, depletion of Vps13D causes the accumulation of mitophagy intermediates (top right) that are polyubiquitinated, coated with Ref(2)p (*Drosophila* homolog of p62), engaged with a stalled phagophore (labeled by Atg8 which is the *Drosophila* homolog of LC3), and lacking in matrix proteins likely due to rupture. **B)** Quantification of successful eclosion frequency of pupae with the indicated RNAi driven by pan-neuronal driver nSyb-Gal4. n.s. indicates no significant difference, *** indicates p<0.001, and **** indicates p<0.0001 based on Chi square test of two compared genotypes indicated.

In Vps13D depleted neurons, loss of *Atg5* led to only minor (1.2 fold increase), but still significant, changes in the percentage of neurons containing mitophagy intermediates (**Fig 6C and 6D**). In contrast, loss of *Atg5* in Drp1 depleted neurons led to a dramatic increase (6.8 fold) in the percentage of neurons that contained Ref2p+ mitophagy intermediates (**Fig 6E and 6F**). This trend was similarly observed when we knocked down *Atg5* with RNAi in *drp1* mutant neurons (**S10 Fig**). These observations support the interpretation suggested by the mCherry-GFP-Atg8A reporter data that mitophagy is both induced and completed in Drp1 depleted neurons, however fails to complete in Vps13D depleted neurons (**Fig 7A**).

The Ref(2)p+ mitophagy intermediates that become trapped in *Atg5* null; Drp1 depleted neurons, and similarly in *drp1* mutant animals expressing *Atg5* RNAi, showed some significant differences from the intermediates lacking matrix that have been described thus far: they are smaller in size, and largely contain mitoGFP (**S10 Fig**). Therefore, the lack of mitochondrial integrity and loss of matrix components in mitophagy intermediates is not a default consequence of impaired mitophagy completion. We interpret that Drp1 loss leads to the induction of two distinct classes of mitophagy intermediates. The first retain their matrix and are usually undetectable due to rapid destruction by autophagy. The second, less abundant, class of intermediates show a loss in matrix components. Similarly to the intermediates that accumulate in Vps13D mutants, these mitochondria may be stalled for mitophagy completion, since their numbers do not increase upon loss of Atg5.

### Functional importance of mitochondrial dynamics and mitophagy in neurons

We expected that the failed clearance of mitophagy intermediates in Vps13D and Atg5; Drp1 depleted neurons should have negative consequences for neurons and the overall health of animals. While we observed no evidence for apoptosis, we noticed that animal survival negatively correlates with the accumulation of mitophagy intermediates. Pan-neuronal knockdown (via nSyb-Gal4) of Vps13D leads to severe lethality in flies; only 5% of animals successfully eclosed from their pupal cases. In comparison, knockdown of Drp1 using the same Gal4 driver leads to a successful eclosion rate of 84% (**Fig 7B**). Simultaneous neuron-specific knockdown of the core autophagy protein Atg5 along with Drp1 causes a significant decrease in eclosion rate (11%), which is much lower than in the cases of knocking down either protein alone (**Fig 7B**). These observations are consistent with a two-hit model (**Fig 7A**) for toxicity: loss of Drp1 alone is only mildly toxic because induction of mitophagy can clear defective mitochondria. In addition, loss of autophagy alone is not toxic because neurons undergo only low levels of basal mitophagy in the absence of any additional stressors. However, scenarios that both induce mitochondrial damage and impair clearance lead to strong lethality. Impairments in Vps13D function can lead to dual defects in damage and defective clearance, providing an attractive explanation for the accelerated neurological pathophysiology associated with patients harboring mutations in this gene [30–32].

## Discussion

Since the discovery of a role for PD associated proteins Parkin and PINK1 in removal of damaged mitochondria via mitophagy, there has been a sustained effort to understand the importance of mitophagy in the maintenance of neuronal health and function. However, discoveries made from mitophagy assays performed in cultured cells have been difficult to verify *in vivo* due to the lack of a robust means of detecting, experimentally inducing, or blocking mitophagy in neurons. Detection of mitophagy, even in cultured cells, generally requires an increase in mitochondrial damage from a stress to drive mitochondria into mitophagy, or the blockage of mitophagy to visualize mitochondria stalled in the pathway. Our data suggest that Vps13D depletion in neurons does both of these, which leads to the accumulation of stalled mitophagy intermediates (**Fig 7A**). First, loss of Vps13D disrupts fission; this stress leads to the induction of mitophagy. Second, loss of Vps13D disrupts mito-phagophore elongation; this leads to the accumulation of stalled mitophagy intermediates. Lastly, stalled mitophagy intermediates in Vps13D depleted neurons are of compromised integrity due to a lack of matrix content, which is potentially due to rupture. The identification of these intermediates and their readily visualizable features (including their large size) opens up future opportunities to study the molecular mediators and mechanism of neuronal mitophagy.

While the genetic manipulations we performed here may represent extreme experimental conditions, the two-hit defect of accumulated mitochondrial damage combined with diminished capacity for clearance is considered to occur naturally during aging and to be accentuated by conditions of stress and/or genetic mutations in many neurological diseases [47]. Our ability to dissect these causally related defects in the study of Vps13D should promote new inroads into understanding the pathophysiology of neurological disease.

### Mitochondrial fission in neurons

At face value, the finding that mitophagy can occur when Drp1 function is impaired may be surprising, since several studies have reported an essential role for mitochondrial fission in mitophagy [20–22]. In addition, Drp1 and Vps13D mutants were reported to show equivalent defects in mitochondrial clearance in *Drosophila* intestinal cells [33]. However, other studies have documented fission-independent mitophagy [23,48]. We propose that differences in these observations may relate to differences between mitophagic clearance and quality-control mitophagy, and differences in cell types. Fission may be important for breaking down a highly fused tubular mitochondrial network during mitophagic clearance [49]. However, neurons are highly reliant on their mitochondria and are not expected to remove all of their mitochondria; instead, mitophagy of only the most damaged mitochondria for quality-control is expected to be critical for long term maintenance of a healthy pool of mitochondria. Our findings imply that neurons deficient for Drp1 and/or Vps13D function lead to mitochondrial damage that induces mitophagy. While stalled mitophagy intermediates have been observed in Drp1-ablated Purkinje cells in the mouse cerebellum [24], the interpretation of the existence of these intermediates differed for ours, since it was inferred that mitophagy was blocked. In contrast, our findings indicate that mitophagy is induced and completed in Drp1 mutant *Drosophila* neurons, while a small and unique population of mitochondria lacking matrix (which may be ruptured), appear stalled as mitophagy intermediates.

How could disruption of mitochondrial fission induce mitophagy in neurons? Previous studies have shown that conditional genetic ablation of Drp1 in either mouse cerebellar or hippocampal neurons leads to oxidative stress with varying outcomes [24,50] (cell lethality or the induction of Nrf2-dependent stress response, respectively). Accumulation of mitochondrial reactive-oxygen species (ROS) has previously been shown to be a trigger of mitophagy [51]. It will therefore be interesting future work to investigate the potential contributions of mitochondrial ROS in Vps13D mutant neurons in animals and ataxia patients, as administration of antioxidant compounds was capable of suppressing the neurodegenerative phenotype associated with Drp1 loss in Purkinje cells [24].

We propose that accelerated mitochondrial damage caused by disruptions in fission lead to increased mitophagy in neurons as an adaptive response required to efficiently degrade damaged organelles. Our results suggest that mitophagy intermediates pose a cellular threat, as shown by the severe lethality caused by either Vps13D loss in neurons and the combined depletion of Drp1 and Atg5 (**Fig 7B**). Other reports have documented increased induction of autophagy upon disruptions of the balance of mitochondrial fission and fusion in neurons including *Drosophila* models of Charcot-Marie Tooth Type 2A syndrome (CMT2A) [52], and rodent models of autosomal dominant optic atrophy [53].

### Stalled mitophagy intermediates lose matrix components

The stalled mitophagy intermediates in Vps13D depleted neurons were lacking in endogenous matrix proteins PDH and Hsp60 (**Fig 2**), in addition to exogenously expressed mitoGFP and Idh3b-HA. One possible explanation for the loss in matrix components could be a failure in mitochondrial import due to prolonged mitochondrial depolarization. However, we think this explanation is unlikely, since the intermediates still contain peripherally associated IMM proteins (ATP5A and ATPβ), which are similarly dependent upon mitochondrial import machinery. The explanation of disrupted import also requires that the lost mitochondrial proteins (ie, PDH and Hsp60) have a short enough half-life for depletion via failed import to be observable. Previous proteomics measurements in adult flies demonstrated that matrix and IMM mitochondrial proteins have similarly long half-lifes (ie, the half-life of Hsp60 was estimated to be ~10.5 days) with high variability for different proteins [54,55]. Because we see the lack of these proteins in mitochondria of 4–6 day-old larvae, we believe an acute event like rupture, consistent with the physical membrane breaks observed by EM, is a more likely reason for the loss in soluble matrix proteins. Another non-mutually exclusive possibility is that soluble matrix proteins could be rapidly degraded by activation of mitochondrial proteases in these stalled mitophagy intermediates. However, mitochondrial proteases are generally thought to act in quality control of misfolded and/or damaged proteins, and not in non-selective wholesale degradation [56].

Other reports have demonstrated rupture of the outer mitochondrial membrane (OMM) during toxin-induced bulk mitophagy in cultured cells, which enables the phagophore to interact with autophagy receptors, such as prohibitin 2, in the IMM [57–59]. In contrast, our observations suggest rupture of both the OMM and IMM membranes. Mitochondrial swelling and rupture has been observed in many cell types undergoing various forms of cell death, which is typically mediated by a so-called mitochondrial permeability transition pore (MPTP) [60]. However, our ultrastructural analysis and cleaved caspase staining did not reveal any dying cells in the VNC at the larval stages when we deplete Vps13D from neurons. We also note that the ruptured mitochondria lacking matrix do not appear vacuolated, as has been described in other neurodegenerative disease models [61–63].

We hypothesize that the strong pupal lethality caused by pan neuronal depletion of Vps13D may be a consequence of mitochondrial rupture. While we did not observe direct evidence of neuronal apoptosis, we consider that mitochondrial DNA and oxidized mitochondrial proteins are potent inducers of innate immune responses [64], and neuro-immune interactions are increasingly being recognized as important factors in neurodegenerative disease [65,66]. Therefore, there is interesting work ahead to understand the pathology that evolves following Vps13D loss.

### Vps13 proteins and cellular function

The stalling of phagophores that we have observed in Vps13D depleted neurons appears most similar to phenotypes that have been described for *Atg2* mutants in yeast and flies [45,67,68]. Importantly, Atg2 contains a unique Chorein_N domain that exists only in Atg2 and in members of the Vps13 protein family. Recent work has demonstrated that this domain, from the Vps13 family member in *C*. *thermophilum*, can directly function as a phospholipid transporter *in vitro* [69–71]. Taken together, these observations raise an interesting hypothesis that Vps13D, and potentially other Vps13 family members, function similarly to Atg2 to enable phospholipid transport critical for phagophore elongation in neurons. This idea is supported by previous findings that the *Dictyostelium* homolog of Vps13A/C is required for proper autophagic flux [72]. Important future questions include: do all Vps13 family members have a similar function phagophore elongation? And are these functions separable from each other and that of Atg2? One idea is that these proteins may be used for similar functions but in different contexts, for instance mitophagy versus non-selective autophagy. Interestingly, loss of Vps13D in larval fat bodies does not disrupt starvation-induced autophagy [33] or most autophagy in neurons (**Fig 5A and 5B**), suggesting a specialized function in some but not all forms of autophagy.

Vps13D’s role in mitochondrial fission likely relies on its function in inter-organelle phospholipid transport, since contacts between the endoplasmic reticulum (ER) and mitochondria have been shown to play a direct role in fission [73]. While this manuscript was under review, further details regarding the cellular localization of Vps13D in cultured cells was reported [74]. Guillén-Samander and colleagues demonstrated that Vps13D localizes to ER/mitochondria and ER/peroxisomal contact sites through associations with mitochondrial/peroxisomal protein Miro and the ER protein Vap. Other work also places Vps13D as a regulator of ER-mitochondrial contact sites [75], but a mechanistic understanding of Vps13D’s role in mitochondrial dynamics is still poorly understood. Proper phospholipid composition in mitochondrial membranes, which requires ER/mitochondrial lipid exchange, modulates Drp1 function [76]. Recent reports indicate the importance of phosphatidylinositol-4-phosphate (PI4P) in mitochondrial fission [77,78]. While little is known about the specific lipid transport activity of Vps13D, previous work has indicated that the singular yeast Vps13 protein can bind PI4P [79]. These observations provide an attractive model for how low levels of Vps13D can lead to impairments in mitochondrial fission. In contrast, it remains unclear how Vps13D loss leads to impaired elongation of the phagophore. It has been recognized that the localization of other Vps13-family proteins is mediated by organelle-specific adaptor proteins [28]. We therefore hypothesize that yet undiscovered interactions mediate recruitment of Vps13D to the phagophore, where it may mediate the transfer of lipids to fuel the growth of the phagophore membrane.

Do the other Vps13 family members share Vps13D’s dual roles in mitochondrial fission and phagophore elongation? Morphological defects in mitochondria have been noted in cell lines lacking Vps13A [80], and cells lacking Vps13C [81]. Loss of Vps13D is uniquely lethal in animal models [30,33,82], which we hypothesize may reflect an essential role for Vps13D in mitochondrial fission in all cell types. In neurons, fission defects lead to an induction of mitophagy, which reveals Vps13D’s second function in phagophore elongation. With this new method to induce mitophagy in neurons, future work can evaluate whether other Vps13 family members are also required for mito-phagophore elongation.

In conclusion, we have shown that Vps13D is essential for two processes that are critical to general mitochondrial health: mitochondrial fission and mitophagy (**Fig 7A**). This knowledge now establishes a framework for future work to determine whether patient mutations in *VPS13D* differentially affect one or both of these processes, which should lead to a better understanding of disease pathogenesis in patients with mutations in this gene [30–32].

## Materials and methods

### Fly maintenance and Drosophila stocks

Fly stocks were maintained on standard Semi-defined yeast-glucose media. All experiments used feeding (not wandering) 3rd instar larvae which were cultured at 25° in a 12:12h light:dark cycle incubator.

The following strains were used in this study (“BL” indicates a strain from Bloomington Stock Center): *Vps13D* RNAi (BL #38320), *luciferase* RNAi (Control RNAi) (BL #31603), D42-Gal4 (BL #8816), Elav-Gal4 (BL #458), *Vps13D* mutants (*Vps13D*^*11101*^ from BL #56282, and *Df* for *Vps13D* BL #25688), *Atg5* mutant (*Atg5*^*5cc5*^ from G. Juhasz’s lab [46]), *Drp1* RNAi (BL #67160), *Atg5* RNAi (BL #34899), *drp1* mutants (*drp1*^*1*^ from BL #24885; *drp1*^*2*^ from BL #24899; *drp1*^*KG*^ from BL #13510; *Df* for *drp1* BL #7494), UAS-mCherry-GFP-Atg8A (gift from T. Neufeld lab), UAS-mitoQC (received from A. Whitworth lab, [15]), UAS-Idh3a-HA (gift from I. Duncan lab [37]), and nSyb-Gal4 (BL #51635).

An unbiased mixture of both male and female larvae were selected for all experiments unless listed below. Because of the presence of the *Atg5* gene on X-chromosome, experiments in **Fig 6** were exclusively performed in male larvae to achieve homozygosity (*Atg5*^*5cc5*^/y).

### Immunohistochemistry

Third instar larvae were selected based on visual and fluorescent markers, and dissected in ice-cold PBS. Fixation was either done with 4% formaldehyde in PBS for 20 minutes at room temperature, or with undiluted Bouin’s Fixative (Ricca Chemical Cat# 1120–16) for 7 minutes at room temperature. Following fixation, dissected larvae were washed extensively in PBS, followed by PBS-T (PBS with 0.1% Triton X-100) before blocking for at least 30 minutes at room temperature in PBS-T supplemented with 5% normal goat serum. Primary and secondary antibodies were diluted in the same buffer used for blocking. Primary antibodies were incubated overnight at 4°, followed by PBS-T washes. Secondary antibodies were incubated for at least 2 hours at room temperature, followed by PBS-T washes. Following staining, filleted larvae were mounted using Prolong Diamond mounting media (Life Technologies).

The following primary antibodies were used in this study: anti ATP5A* at 1:1000 (Abcam #ab14748), anti dsRed at 1:500 (Takara #632496), anti Polyubiquitin (“PolyUb”) at 1:50 (FK1) (Enzo Life Sciences #pw8805), anti Ref(2)p at 1:500 (Abcam #ab178440), anti Atg8 (GABARAP)* at 1:500 (Cell Signaling Technology #13733S), anti GFP at 1:1000 (Aves Labs #GFP1020), anti GFP at 1:1000 (Life Technologies #A6455), anti GFP** at 1:500 (Life Technologies #A-11120), anti PDH (PDH-e1ɑ)** at 1:200 (Abcam #ab110334), anti Multi Ubiquitin (“Ub/PolyUb”) at 1:200 (FK2) (MBL #D058-3), anti Elav at 1:100 (Developmental Studies Hybridoma Bank #Rat-Elav-7E8A10), anti HA at 1:500 (Sigma #H6908), anti Hsp60** at 1:100 (Cell Signaling Technologies #4870), and anti ATPβ at 1:500 (Abcam #ab14730). Antibodies designated with the (*) symbol were only used in conditions in which tissue was fixed with Bouin’s Fixative due to dramatically better staining in this fixation condition, whereas antibodies labeled with the (**) symbol were only used in conditions in which tissue was stained with 4% formaldehyde due to dramatically better staining in this fixation condition. Any antibodies not designated with those symbols worked well in both fixation conditions. The ubiquitin antibodies FK1 (PolyUb) and FK2 (Ub/PolyUb) showed similar localization, but were used interchangeably based on compatibility with other antibodies in particular staining conditions.

The following secondary antibodies (all derived from goat, diluted 1:1000) were used (all from Life Technologies): anti Rabbit (Alexa Fluor 405/488/568/647), anti Mouse IgG1 (Alexa Fluor 568/647), anti Mouse IgG2a (Alexa Fluor 488), anti Mouse IgG2b (Alexa Fluor 488/647), anti IgM (Alexa Fluor 568), and anti Rat (Alexa Fluor 488/647).

### Electron microscopy

Third instar larvae were dissected in ice cold PBS then fixed with 3.2% paraformaldehyde, 1% glutaraldehyde, 1% sucrose and 0.028% CaCl_2_ in 0.1 N sodium cacodylate (pH 7.4, overnight, 4 ^o^C). Samples were then postfixed with 0.5% OsO_4_ (1h, RT) then half-saturated aqueous uranyl acetate (30 min, RT), dehydrated in graded series of ethanol and embedded using Spurr Low-Viscosity Embedding Kit (EM0300, Sigma-Aldrich) according to the manufacturer’s instructions. Ultrathin 70-nm sections were stained in Reynold’s lead citrate and viewed at 80kV operating voltage on a JEM-1011 transmission electron microscope (JEOL) equipped with a Morada digital camera (Olympus) using iTEM software (Olympus).

### Imaging and quantification

Confocal images were collected on an Improvision spinning disk confocal system, consisting of a Yokagawa Nipkow CSU10 scanner, and a Hamamatsu C1900-50 EMCCD camera, mounted on a Zeiss Axio Observer with 63X (1.4NA) oil objectives or a Leica SP5 Laser Scanning Confocal Microscope with a 63x (1.4NA) oil objective. Images in **S4 Fig** of individual mitophagy intermediates were subjected to deconvolution using Leica LAS AF deconvolution module. Similar settings were used for imaging of all compared genotypes and conditions. Volocity software (Perkin Elmer) was used for intensity measurements and quantification of all confocal data. All images from conditions of pan-neuronal manipulations (D42-Gal4 or Elav-Gal4) were taken and quantified from the dorsal midline motoneurons (approximately segments A3-A7).

For live larval imaging, third instar larvae were dissected in room temperature HL3 [83] with 0.45mM Ca^2+^, as previously described [84]. Snapshots of neurons from larval VNCs were captured with a 63x objective lens using the Improvision spinning disk confocal system. All images acquired in conditions that were normalized together were obtained with identical microscope settings.

To quantify the mean intensities of mitochondrial proteins in PolyUb+ or Ref(2)p+ objects (**Figs 1C, 2 4B and 4E**) (mitoGFP, ATP5A and PDH), the average intensity of mitochondrial protein staining was collected from >500 individual *bona fide* mitochondria that were PolyUb-. Objects with intensities above the 2.5% percentile of this population (indicated by the semi-transparent rectangles) were scored as positive.

To quantify the phagophore engulfment of mitochondria (**S4 Fig**), separate segmented populations were defined based on intensity using the Volocity software for PolyUb and Atg8 for each mitophagy intermediate, along with a third population which represented the voxel space occupied by the overlap of these two populations. The overall accumulation of Atg8 staining (volume (um^3^) multiplied by the intensity (A.U.)) was plotted against the voxel overlap of the two populations (Atg8 and PolyUb) to test for a correlation. The expectation is that there would be a positive correlation between the size of the phagophore and the overlap of Atg8 and PolyUb signal if the phagophore was successfully engulfing the PolyUb+ mitochondria.

To quantify the mCherry-only signal from live imaging of neurons expressing mCherry-GFP-Atg8A (**Figs 5 and S8**), neurons with reporter expression were identified by their diffuse, cytoplasmic localization of the reporter (typically in the GFP channel). For each neuron, a selection algorithm was designed in Volocity to select bright, red-only signal, and similarly applied to all individual neurons. The total mCherry-only signal, accounting for the brightness and total amount (sum pixel intensity), was normalized to 1 for control conditions that were carried out in parallel for each experiment. Conditions that were compared were subjected to the same imaging settings. If a neuron did not contain any mCherry-only signal, it was excluded from comparison to other conditions, but the % of neurons lacking mCherry-only signal was noted for each condition.

To quantify the % of polyubiquitinated mitochondria with phagophores (in text and **S9 Fig**), ATP5A+/PolyUb+ objects were individually assessed for the presence of recognizable Atg8 staining.

To quantify the % of neurons containing mitophagy intermediates (**Fig 6**), mitophagy intermediates were identified as Ref(2)p+ objects that contained ATP5A, and were confirmed to be neuronal based on proximity to labeling of the neuronal nuclear marker Elav. The number of neurons containing one or more mitophagy intermediates were counted from the entirety of motoneurons in the dorsal midline from each ventral nerve cord.

Volume measurements of mitophagy intermediates (**S10 Fig**) were performed in Volocity, and based off of the volume of Ref(2)p+, ATP5A+ objects.

### Eclosion assay

Pupae of the proper genotype (based on morphology and fluorescence) were counted 8 days following egg laying, and counted pupae were tracked for a total of 7 days. Successful eclosion was counted as complete exit from the pupal case, as flies that died while partially eclosed were counted as failed.

### Statistics

All statistical methods utilized are listed in the Figure legends, and in almost all scenarios (except for correlation test in **S4 Fig** and eclosion assays in **Fig 7B**). Two-tailed unpaired T-tests assuming parametric distributions were used to compare different conditions. All error bars represent standard error of the mean (SEM), and individual data points are included to indicate the distribution of the data. The statistical significance of Eclosion Assays were determined using a Chi-Square Test to compare two individual genotypes at a time. Sample sizes were determined based on previous literature.

## Supporting information

S1 Fig *Vps13D* mutant larvae contain mitophagy intermediates in neurons, similar to *Vps13D* RNAi expressing animals. Representative images of dorsal midline motoneurons in early second instar larval VNCs from a *Vps13D* heterozygous (*Vps13D*^*11101*^*/+)* background (top panels) or *Vps13D* mutant (*Vps13D*^*11101*^*/Df)* background (bottom panels). Arrowheads highlight mitophagy intermediates. The insets show higher magnification image of single neuronal cell bodies (identified by the dashed box). **A)** Tissues were stained for the neuron-specific transcription factor Elav (cyan), autophagy receptor protein Ref(2)p (red), and mitochondrial protein ATP5A (yellow). Arrowheads highlight mitophagy intermediates (Ref(2)p+/ATP5A+).Scale bars = 10μm, 2μm. **B)** Tissues were stained for neuron-specific transcription factor Elav (cyan), phagophore marker Atg8A/B (red), and mitochondrial IMM protein ATP5A (yellow). Arrowheads highlight mitophagy intermediates engaged with a phagophore (Atg8+/ATP5A+). Scale bars = 10μm, 2μm.
(TIF)

S2 Fig Additional mitochondrial matrix marker lacking in PolyUb+ objects in Vps13D depleted neurons. Representative images of single motoneuron cell bodies which co-express indicated RNAi via the D42-Gal4 driver, together with the full-length tagged matrix protein Idh3b-HA (UAS-Idh3b-HA). Idh3b is detected based on antibody staining for the HA tag (cyan). White arrowheads indicate PolyUb+ (red) mitophagy intermediates that lack Idh3b-HA. Scale bar = 2μm.
(TIF)

S3 Fig Additional examples of mitophagy intermediates from EM analysis of Vps13D depleted neurons. **A)** Two examples of larval neurons expressing *Vps13D*-RNAi which contain mitochondria engaged with a phagophore. In the lower magnification images on the left side, mitochondria (M) that are not engaged with a phagophore have compact and electron dense cristae. In comparison, the phagophore-associated mitochondria (M_p_) have cristae that appear less compactly organized. High magnification image of mitophagy intermediates (dashed black box) are shown to the right. Arrowheads indicate the phagophore. **B)** Two additional examples of mitochondrial rupture by phagophore-associated mitochondria (M_p_) in *Vps13D*-RNAi neurons. Arrowheads indicate the phagophore, while arrows indicate locations on the mitochondria lacking IMM and OMM. Scale bars indicated in images.
(TIF)

S4 Fig Partial phagophores associated with mitophagy intermediates in Vps13D depleted neurons. **A)** Representative images of individual mitophagy intermediates engaged with a range of phagophore sizes (smallest to largest from top to bottom). Mitophagy intermediates were stained for mitochondrial marker ATP5A (blue), polyubiquitin (PolyUb) (magenta), and phagophore protein Atg8A/B (green). Left panel shows projected confocal image, and 3D renderings of the projections are shown in the middle and right panels to portray the shape of the engaged phagophore on the mitophagy intermediate. Scale bar = 0.5μm **B)** Quantitative analysis of phagophore engulfment of mitophagy intermediates. The sum of Atg8 staining per mitophagy intermediate (Volume x Intensity) is plotted on the X-axis against the voxel overlap of Atg8 (green) and polyubiquitin (red). Each point represents a single mitophagy intermediate. (n.s. correlation indications non-significance in Pearson’s Correlation test, n = 194 XY pairs collected from 5 larvae VNCs, p = 0.3). If the phagophore was successfully engulfing the mitochondria, we expect there would be a positive correlation between the size of the phagophore and the overlap with PolyUb.
(TIF)

S5 Fig Further characterization of *drp1* mutant motoneurons. **A)** Representative images of individual dorsal midline motoneuron cell bodies which co-express the indicated RNAi together with mitoGFP (greyscale) driven by the pan-motoneuron driver D42-Gal4. RNAi depletion of either Vps13D (BL# 38320) or Drp1 (BL# 67160) leads to enlarged mitochondrial morphology. Scale bar = 2μm. **B)** Representative images of individual dorsal midline motoneuron cell bodies from the indicated genotypes which express mitoGFP (greyscale) driven by the pan-motoneuron driver D42-Gal4. Enlargement of mitochondrial morphology is most severe and similar to *Drp1* RNAi condition in the *drp1*^*KG*^*/Df* genotype (bottom). Morphological enlargement is more pronounced in *drp1*^*2*^*/drp1*^*KG*^ compared to *drp1*^*2*^*/drp1*^*2*^ genotype. Scale bar = 2μm. **C)** Representative images of motoneurons in the larval VNC of indicated *drp1* mutant which express mitochondrial marker mitoGFP (cyan) via the D42-Gal4 driver, which is stained for Ref(2)p (red) and ATP5A (yellow). Arrowheads highlight an example of a mitophagy intermediates lacking mitochondrial matrix protein marker (Ref(2)p+/ATP5A+/mitoGFP-). Scale bar = 2μm.
(TIF)

S6 Fig mitoQC marker does not exclusively label mitochondria or consistently label mitophagy intermediates in larval motoneurons lacking Vps13D. **A)** Representative images of individual dorsal midline motoneuron cell bodies co-expressing the indicated RNAi and UAS-mitoQC via the D42-Gal4 driver. Tissue was fixed and stained with antibodies against RFP to recognize mitoQC reporter (red) and ATP5A (yellow) to label the mitochondria. While mitoQC concentrates on mitochondria in both conditions, it also localizes to the cytoplasm. Scale bar = 2μm. **B)** Representative images of individual dorsal midline motoneuron cell bodies which co-express *Vps13D*-RNAi and UAS-mitoQC via the D42-Gal4 driver. Tissue was fixed and stained with antibodies against GFP to recognize mitoQC reporter (cyan) and Ref(2)p (red) to label the mitophagy intermediates. While mitoQC sometimes concentrates on mitophagy intermediates (example #2), it did not consistently label all mitophagy intermediates in Vps13D depleted neurons (see example #1 where there is no observable concentration above background in the Ref(2)p+ object). Scale bars = 5μm, 2μm.
(TIF)

S7 Fig Further analysis of mCherry-GFP-Atg8A autophagy reporter. **A)** Representative images of individual dorsal midline motoneuron cell bodies co-expressing the indicated RNAi and UAS-mCherry-GFP-Atg8A via the D42-Gal4 driver. Tissue was fixed and stained with antibodies against GFP to recognize the tandem-tagged Atg8A reporter (cyan), Ref(2)p (red), and ATP5A (yellow) to label the mitochondria. The reporter localizes to mitophagy intermediates (arrowheads) in *Vps13D* and *Drp1* RNAi conditions, consistent with endogenous Atg8A/B staining. Scale bar = 2μm. **B)** Representative image of live dorsal midline motoneuron co-expressing *Vps13D* RNAi and UAS-mCherry-GFP-Atg8A via the D42-Gal4. Arrowhead highlights the concentration of reporter in the shape of a round object (presumably a mitochondrion, though not labeled in this live imaging experiment). In contrast, the mCherry channel was dominated by bright puncta which did not colocalize with the hypothesized mitophagy intermediates. We interpret that the bright puncta represent accumulation of reporter in autophagolysosomes resulting from basal autophagy independent of the mitophagy intermediates. Scale bar = 2μm.
(TIF)

S8 Fig Control conditions to verify tandem-tagged reporter results in larval motoneurons. **A)** Representative images of live larval motoneurons expressing UAS-mCherry-GFP-Atg8A, expressed via the D42-Gal4 driver, simultaneous with the indicated protein overexpression (OE) or RNAi. White dashed lines indicate the outlines of individual cell bodies. Scale bar = 10μm. **B)** Quantification of the sum pixel intensity of the mCherry-only signal per neuronal cell body (normalized to UAS-Control (UAS-luciferase)). Each point represents a single neuronal cell body, bars represent the mean ± SEM n = 74 cell bodies (for control), n = 89 cell bodies (for Atg1 OE), and n = 37 cell bodies (for *Atg5* RNAi), obtained from 6 larval VNCs per genotype) **** represents p value <0.0001. **C)** Representative images of live larval motoneurons which co-express UAS-mCherry-GFP-Atg8A along with indicated RNAi, via the D42-Gal4 driver, in *drp1* mutants (*drp1*^*2*^*/drp1*^*KG*^*)*. White dashed lines indicate the outlines of individual cell bodies. Scale bar = 10μm. **D)** Quantification of the sum pixel intensity of the mCherry-only signal per neuronal cell body (normalized to Control RNAi in *drp1* mutant). Each point represents a single neuronal cell body, bars represent the mean ± SEM n = 76 cell bodies (for control), and n = 72 cell bodies (for *Atg5* RNAi), obtained from 7 larval VNCs per genotype. **** represents p value <0.0001.
(TIF)

S9 Fig Further analysis and validation of mitophagy blockage in *Atg5* null mutants. **A,B)** Representative images of individual dorsal midline motoneuron cell bodies which express mitoGFP (greyscale in (A), cyan in (B)) driven by the pan-motoneuron driver D42-Gal4 in WT (*w*^*1118*^) (top) and *Atg5* null (*Atg5*^*5cc5*^) animals (bottom). Scale bar = 2μm. **B)** Tissue was stained for the autophagy receptor Ref(2)p (red) and the mitochondrial IMM protein ATP5A (yellow). Arrowhead highlights a mitophagy intermediate which contains both ATP5A and mitoGFP. Scale bar = 2μm. **C)** Representative images of dorsal midline motoneurons which express *Vps13D* RNAi driven by pan-neuronal driver Elav-Gal4 in a WT (*w*^*1118*^) background (top panel) vs. an *Atg5* null (*Atg5*^*5cc5*^) background (bottom panel). Tissue was stained for ubiquitin (Ub/PolyUb, FK2) (cyan), phagophore protein Atg8A/B (red) and mitochondrial protein ATP5A (yellow). Arrowheads highlight polyubiquitinated mitochondria engaged with a phagophore (top panel). Scale bar = 5μm. **D)** Quantification of the % of polyubiquitinated mitochondria that are engaged with a phagophore. Each point represents the total percentage in the VNC from one animal, and bars represent mean ± SEM. (n = 5 for each condition, each containing >50 polyubiquitinated mitochondria). **** indicates p<0.0001.
(TIF)

S10 Fig Mitophagy intermediates in conditions of combined Drp1 and Atg5 loss. **A)** Histograms depicting the distribution of the volume (μm^3^) of mitophagy intermediates (Ref(2)p+ mitochondria) in fission-deficient conditions in WT and *Atg5* mutant backgrounds. Top histogram represents conditions of *Drp1* RNAi expression (n = 20 mitophagy intermediates for *Drp1* RNAi condition (grey bars), and n = 136 mitophagy intermediates for *Drp1* RNAi in *Atg5* mutant condition (light blue bars)). Bottom histogram represents *Vps13D* RNAi expression (n = 168 mitophagy intermediates for *Vps13D* RNAi condition (pink bars) and n = 144 mitophagy intermediates in *Vps13D* RNAi condition in *Atg5* mutants (red bars)). Blue shaded box with dashed lines indicates a population of smaller mitophagy intermediates that are revealed in Drp1 depleted neurons only when *Atg5* is lost. In contrast to *Drp1* RNAi neurons, no analogous new population of mitophagy intermediates was revealed when Vps13D was depleted in *Atg5* mutant conditions. **B)** Representative images of dorsal midline motoneurons from the *Atg5* mutants (*Atg5*^*5cc5*^*/y*) which co-express mitoGFP (cyan) and the indicated RNAi driven by the pan-neuron driver elav-Gal4. Closed arrowheads highlight stalled mitophagy intermediates that lack mitoGFP; open arrowheads highlight stalled mitophagy intermediates that contain mitoGFP. Scale bar = 5μm. **C)** Quantification of the % of stalled mitophagy intermediates that contain mitoGFP. Verified Ref(2)p+/ATP5A+ objects were designated as mitoGFP+, as described in Materials and Methods in and in the legend for **Fig 1C**. Points represent the % of mitoGFP+ mitophagy intermediates out of total Ref(2)p+/ATP5A+ mitophagy intermediates in one animal, with n = 5 animals per RNAi condition (each condition contained >160 Ref(2)p+/ATP5A+ mitophagy intermediates). Bars represent mean ± SEM. **** indicates p<0.0001. **D)** Representative images of dorsal midline motoneurons from the *drp1* mutants (*drp1*^*2*^*/drp1*^*KG*^) which co-express mitoGFP (cyan) and the indicated RNAi driven by the pan-motoneuron driver D42-Gal4. Tissue was stained for autophagy receptor Ref(2)p (red) and mitochondrial IMM protein ATP5A (yellow). The dashed box outlines a single Gal4-expressing neuronal cell body that is shown in high magnification in the inset. Arrowheads highlight example mitophagy intermediates (Ref(2)p+/ATP5A+). In the inset of *drp1* mutant expressing *Atg5* RNAi (bottom), open arrowheads highlight mitophagy intermediates that contain mitoGFP, while the closed arrowheads highlight mitophagy intermediates that lack mitoGFP. Scale bars = 10μm, 2μm.
(TIF)
