# Supplementary figures and images for: Mitochondrial fission, integrity and completion of mitophagy require separable functions of Vps13D in Drosophila neurons
Source: PLoS Genet. 2021 Aug 12;17(8):e1009731. doi: 10.1371/journal.pgen.1009731 (PMC8384225; doi:10.1371/journal.pgen.1009731)

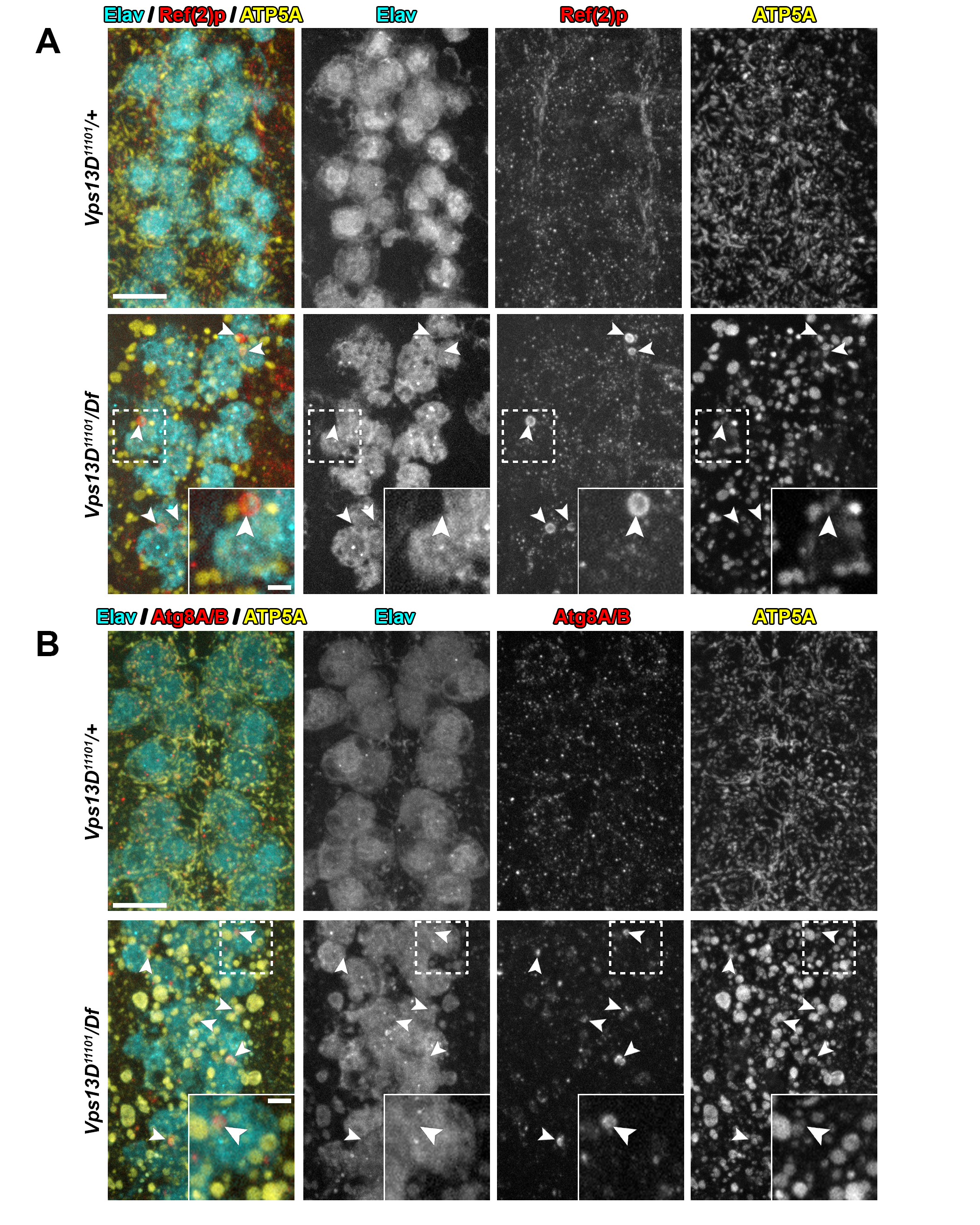

Supplement: S1 Fig — Representative images of dorsal midline motoneurons in early second instar larval VNCs from a Vps13D heterozygous (Vps13D11101/+) background (top panels) or Vps13D mutant (Vps13D11101/Df) background (bottom panels). Arrowheads highlight mitophagy intermediates. The insets show higher magnification image of single neuronal cell bodies (identified by the dashed box). A) Tissues were stained for the neuron-specific transcription factor Elav (cyan), autophagy receptor protein Ref(2)p (red), and mitochondrial protein ATP5A (yellow). Arrowheads highlight mitophagy intermediates (Ref(2)p+/ATP5A+).Scale bars = 10μm, 2μm. B) Tissues were stained for neuron-specific transcription factor Elav (cyan), phagophore marker Atg8A/B (red), and mitochondrial IMM protein ATP5A (yellow). Arrowheads highlight mitophagy intermediates engaged with a phagophore (Atg8+/ATP5A+). Scale bars = 10μm, 2μm. (TIF) [file pgen.1009731.s001.tif]

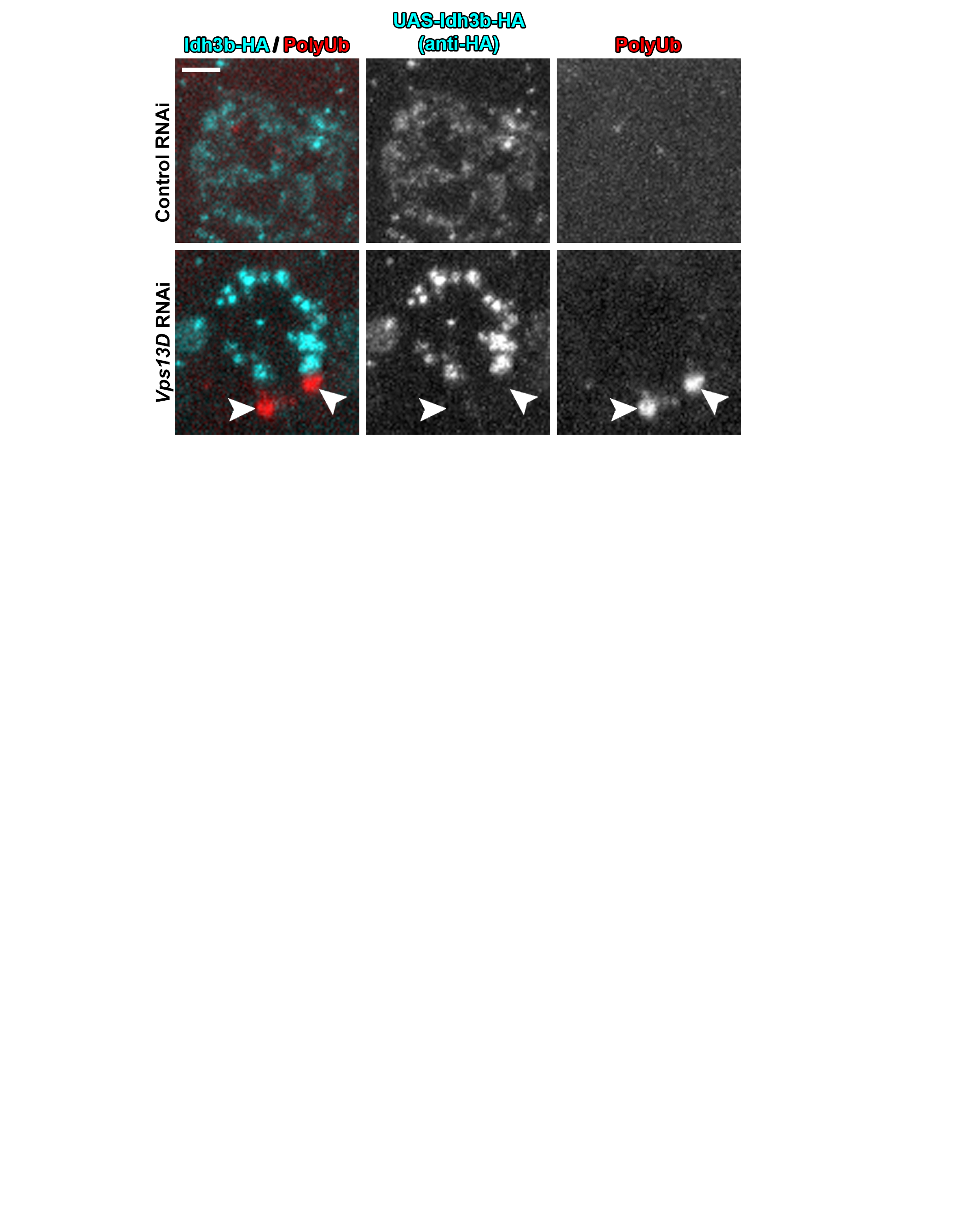

Supplement: S2 Fig — Representative images of single motoneuron cell bodies which co-express indicated RNAi via the D42-Gal4 driver, together with the full-length tagged matrix protein Idh3b-HA (UAS-Idh3b-HA). Idh3b is detected based on antibody staining for the HA tag (cyan). White arrowheads indicate PolyUb+ (red) mitophagy intermediates that lack Idh3b-HA. Scale bar = 2μm. (TIF) [file pgen.1009731.s002.tif]

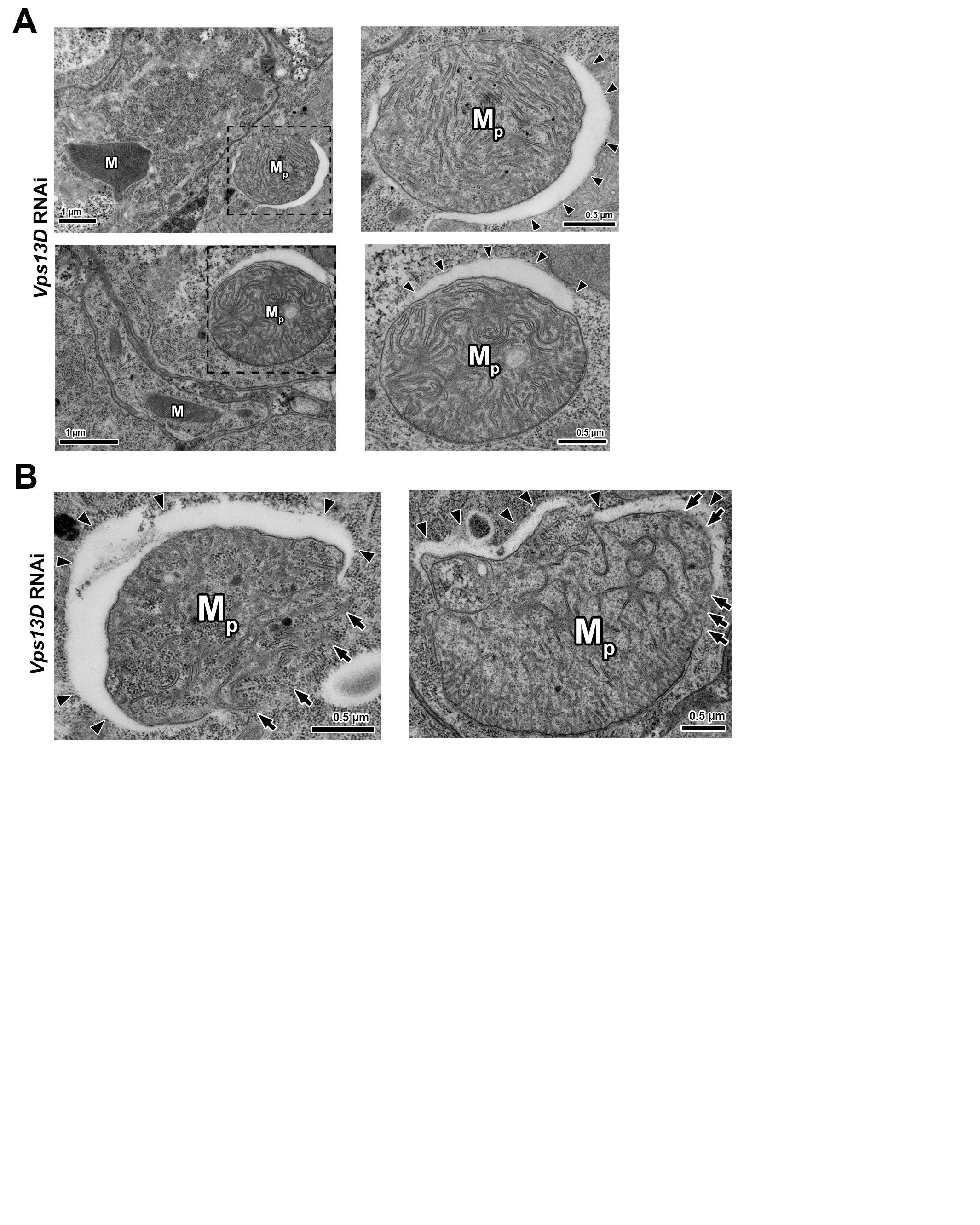

Supplement: S3 Fig — A) Two examples of larval neurons expressing Vps13D-RNAi which contain mitochondria engaged with a phagophore. In the lower magnification images on the left side, mitochondria (M) that are not engaged with a phagophore have compact and electron dense cristae. In comparison, the phagophore-associated mitochondria (Mp) have cristae that appear less compactly organized. High magnification image of mitophagy intermediates (dashed black box) are shown to the right. Arrowheads indicate the phagophore. B) Two additional examples of mitochondrial rupture by phagophore-associated mitochondria (Mp) in Vps13D-RNAi neurons. Arrowheads indicate the phagophore, while arrows indicate locations on the mitochondria lacking IMM and OMM. Scale bars indicated in images. (TIF) [file pgen.1009731.s003.tif]

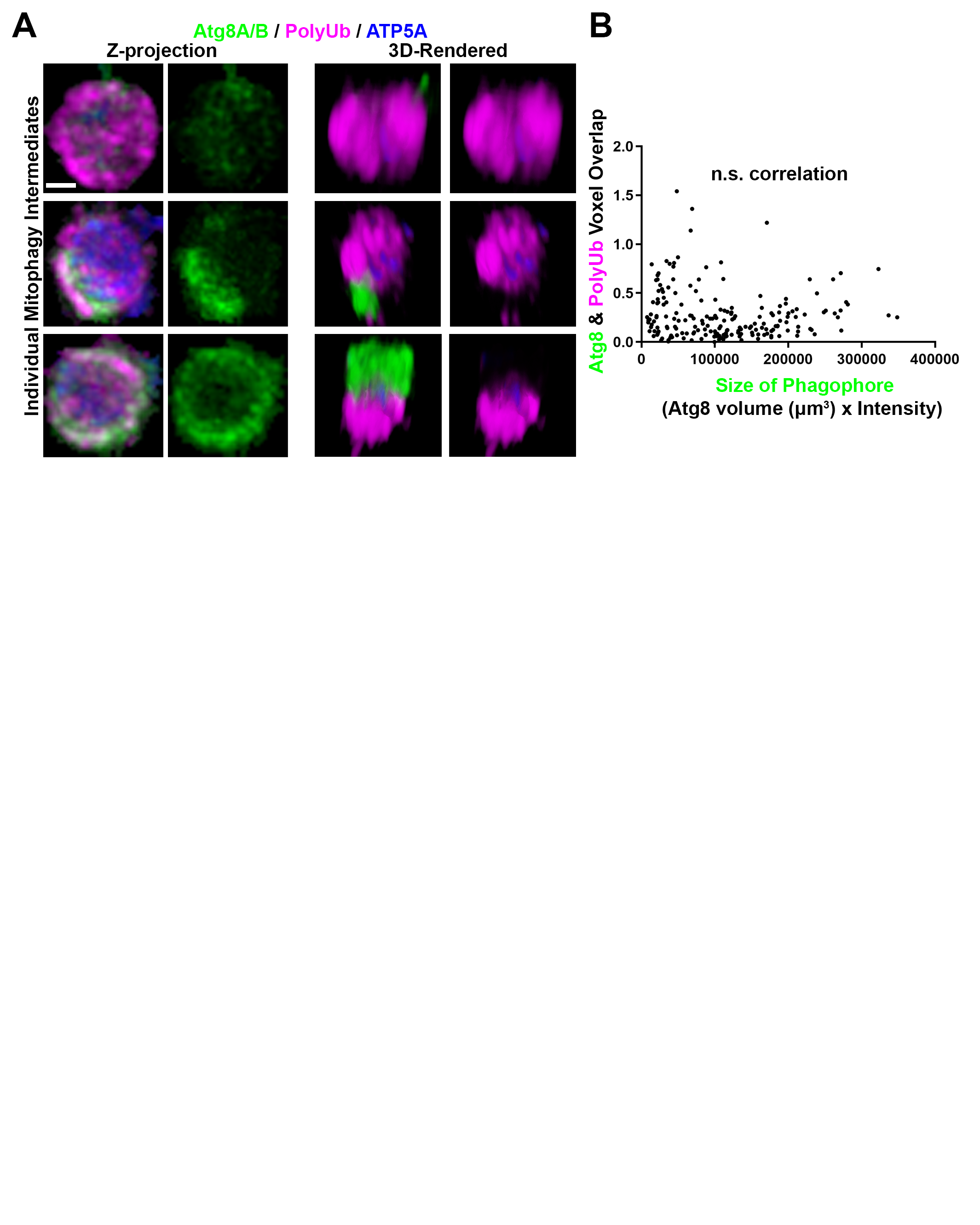

Supplement: S4 Fig — A) Representative images of individual mitophagy intermediates engaged with a range of phagophore sizes (smallest to largest from top to bottom). Mitophagy intermediates were stained for mitochondrial marker ATP5A (blue), polyubiquitin (PolyUb) (magenta), and phagophore protein Atg8A/B (green). Left panel shows projected confocal image, and 3D renderings of the projections are shown in the middle and right panels to portray the shape of the engaged phagophore on the mitophagy intermediate. Scale bar = 0.5μm B) Quantitative analysis of phagophore engulfment of mitophagy intermediates. The sum of Atg8 staining per mitophagy intermediate (Volume x Intensity) is plotted on the X-axis against the voxel overlap of Atg8 (green) and polyubiquitin (red). Each point represents a single mitophagy intermediate. (n.s. correlation indications non-significance in Pearson’s Correlation test, n = 194 XY pairs collected from 5 larvae VNCs, p = 0.3). If the phagophore was successfully engulfing the mitochondria, we expect there would be a positive correlation between the size of the phagophore and the overlap with PolyUb. (TIF) [file pgen.1009731.s004.tif]

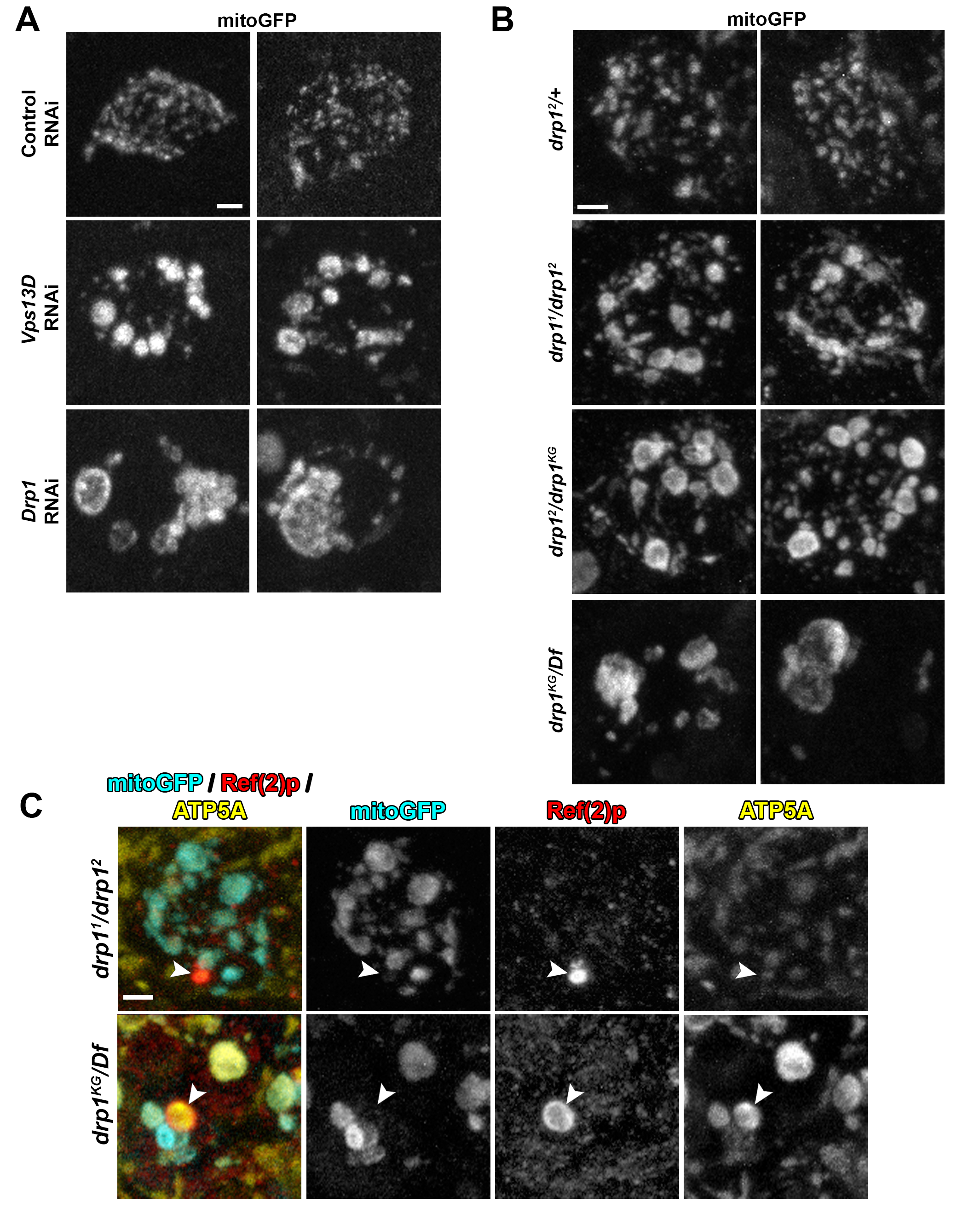

Supplement: S5 Fig — A) Representative images of individual dorsal midline motoneuron cell bodies which co-express the indicated RNAi together with mitoGFP (greyscale) driven by the pan-motoneuron driver D42-Gal4. RNAi depletion of either Vps13D (BL# 38320) or Drp1 (BL# 67160) leads to enlarged mitochondrial morphology. Scale bar = 2μm. B) Representative images of individual dorsal midline motoneuron cell bodies from the indicated genotypes which express mitoGFP (greyscale) driven by the pan-motoneuron driver D42-Gal4. Enlargement of mitochondrial morphology is most severe and similar to Drp1 RNAi condition in the drp1KG/Df genotype (bottom). Morphological enlargement is more pronounced in drp12/drp1KG compared to drp12/drp12 genotype. Scale bar = 2μm. C) Representative images of motoneurons in the larval VNC of indicated drp1 mutant which express mitochondrial marker mitoGFP (cyan) via the D42-Gal4 driver, which is stained for Ref(2)p (red) and ATP5A (yellow). Arrowheads highlight an example of a mitophagy intermediates lacking mitochondrial matrix protein marker (Ref(2)p+/ATP5A+/mitoGFP-). Scale bar = 2μm. (TIF) [file pgen.1009731.s005.tif]

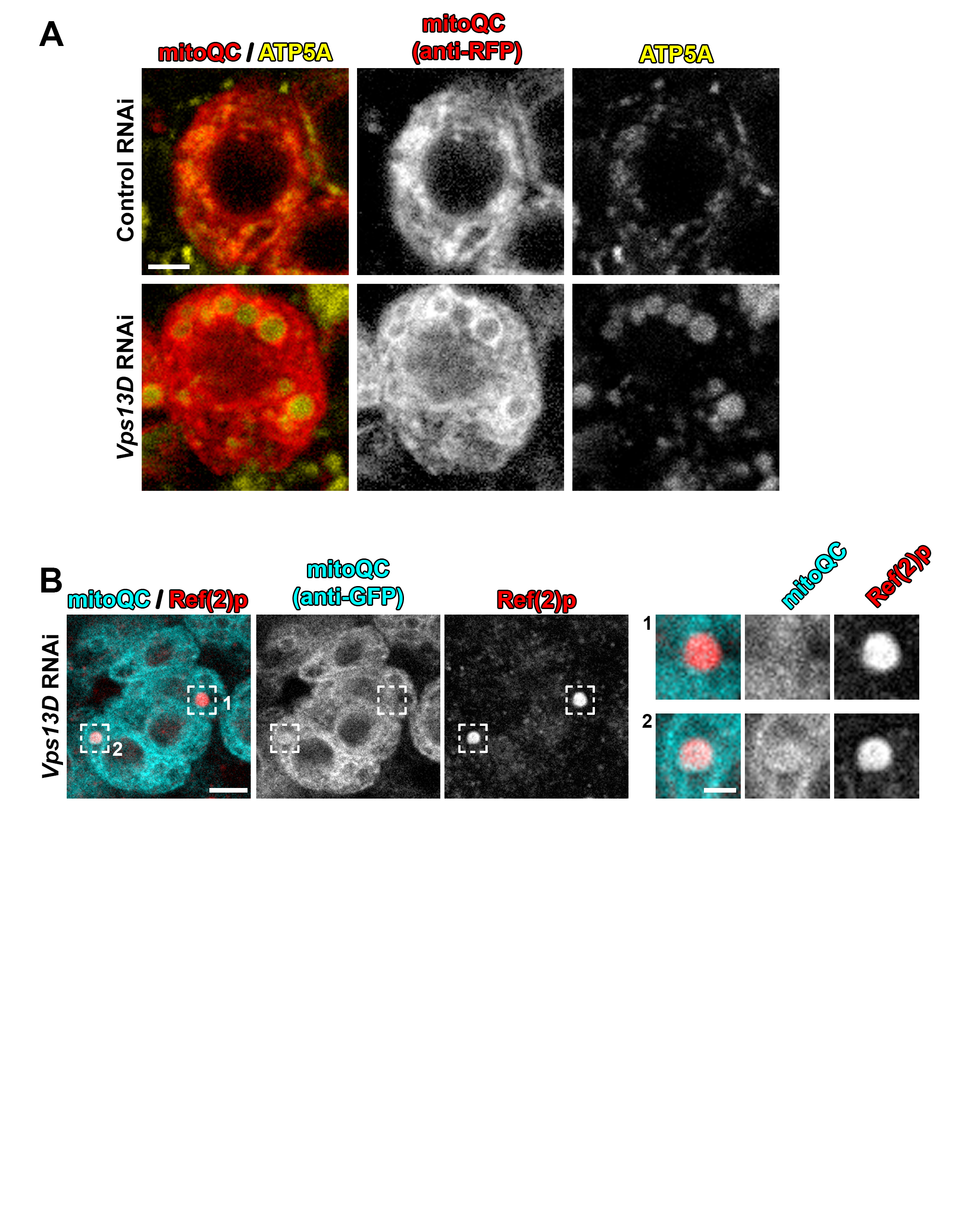

Supplement: S6 Fig — A) Representative images of individual dorsal midline motoneuron cell bodies co-expressing the indicated RNAi and UAS-mitoQC via the D42-Gal4 driver. Tissue was fixed and stained with antibodies against RFP to recognize mitoQC reporter (red) and ATP5A (yellow) to label the mitochondria. While mitoQC concentrates on mitochondria in both conditions, it also localizes to the cytoplasm. Scale bar = 2μm. B) Representative images of individual dorsal midline motoneuron cell bodies which co-express Vps13D-RNAi and UAS-mitoQC via the D42-Gal4 driver. Tissue was fixed and stained with antibodies against GFP to recognize mitoQC reporter (cyan) and Ref(2)p (red) to label the mitophagy intermediates. While mitoQC sometimes concentrates on mitophagy intermediates (example #2), it did not consistently label all mitophagy intermediates in Vps13D depleted neurons (see example #1 where there is no observable concentration above background in the Ref(2)p+ object). Scale bars = 5μm, 2μm. (TIF) [file pgen.1009731.s006.tif]

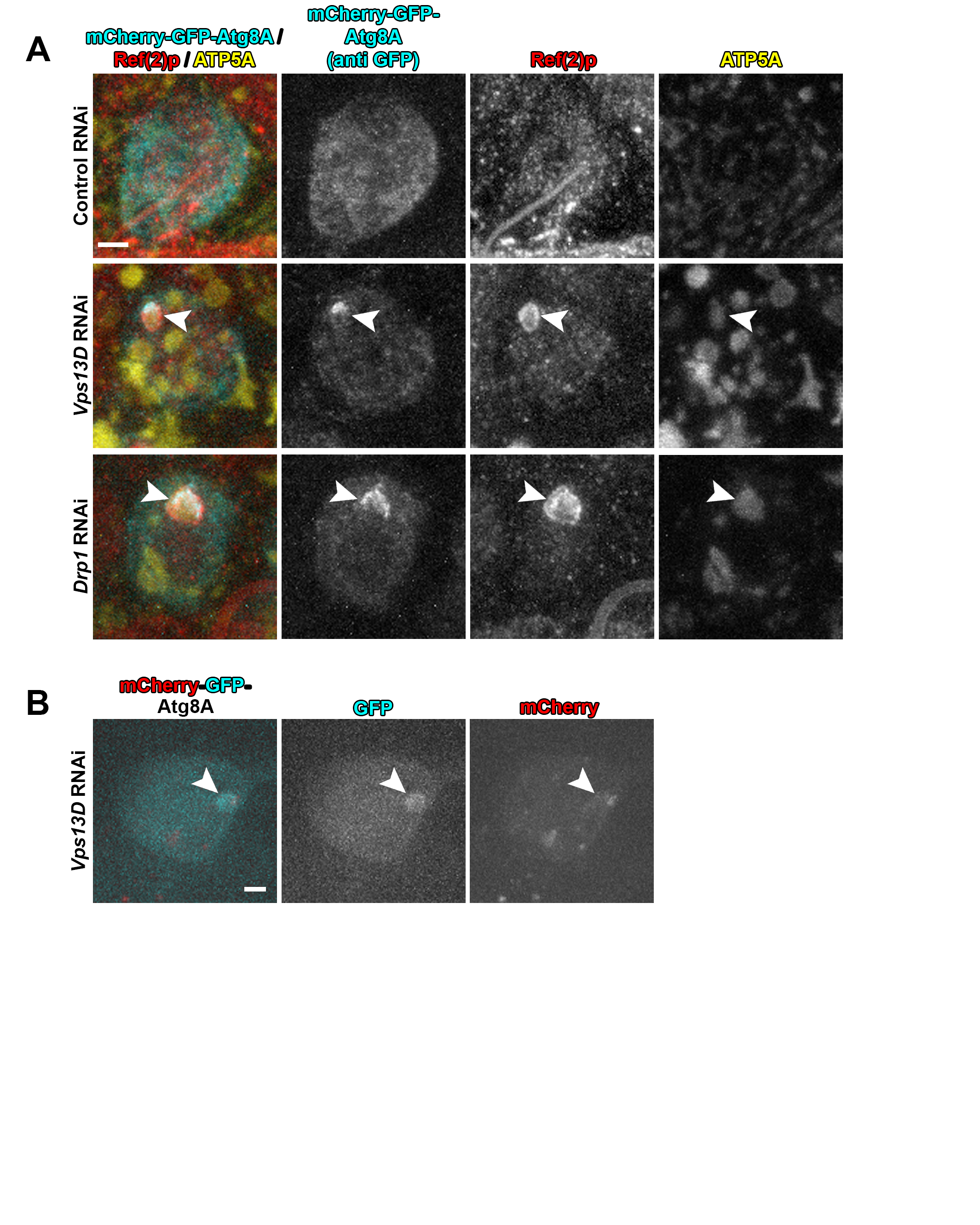

Supplement: S7 Fig — A) Representative images of individual dorsal midline motoneuron cell bodies co-expressing the indicated RNAi and UAS-mCherry-GFP-Atg8A via the D42-Gal4 driver. Tissue was fixed and stained with antibodies against GFP to recognize the tandem-tagged Atg8A reporter (cyan), Ref(2)p (red), and ATP5A (yellow) to label the mitochondria. The reporter localizes to mitophagy intermediates (arrowheads) in Vps13D and Drp1 RNAi conditions, consistent with endogenous Atg8A/B staining. Scale bar = 2μm. B) Representative image of live dorsal midline motoneuron co-expressing Vps13D RNAi and UAS-mCherry-GFP-Atg8A via the D42-Gal4. Arrowhead highlights the concentration of reporter in the shape of a round object (presumably a mitochondrion, though not labeled in this live imaging experiment). In contrast, the mCherry channel was dominated by bright puncta which did not colocalize with the hypothesized mitophagy intermediates. We interpret that the bright puncta represent accumulation of reporter in autophagolysosomes resulting from basal autophagy independent of the mitophagy intermediates. Scale bar = 2μm. (TIF) [file pgen.1009731.s007.tif]

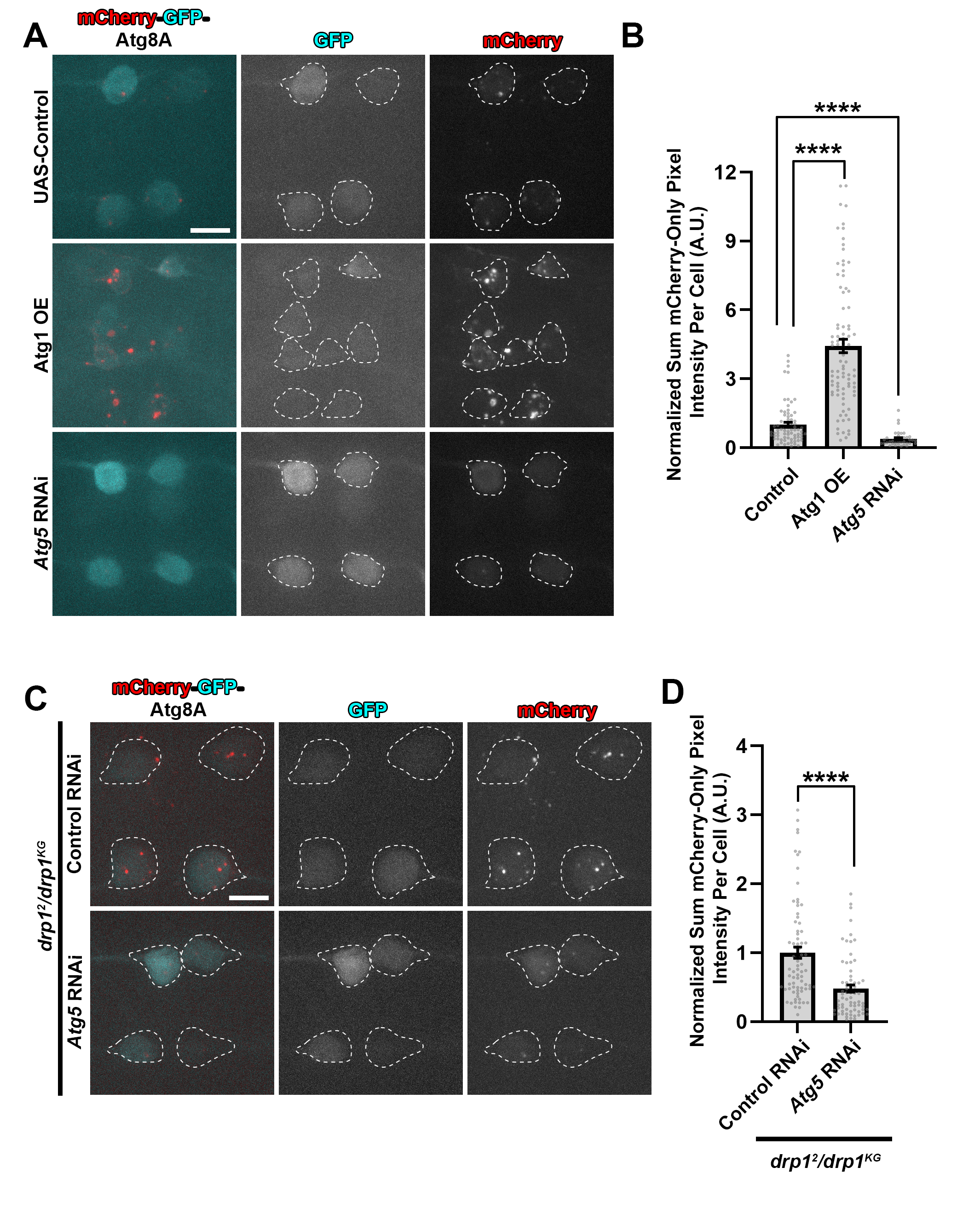

Supplement: S8 Fig — A) Representative images of live larval motoneurons expressing UAS-mCherry-GFP-Atg8A, expressed via the D42-Gal4 driver, simultaneous with the indicated protein overexpression (OE) or RNAi. White dashed lines indicate the outlines of individual cell bodies. Scale bar = 10μm. B) Quantification of the sum pixel intensity of the mCherry-only signal per neuronal cell body (normalized to UAS-Control (UAS-luciferase)). Each point represents a single neuronal cell body, bars represent the mean ± SEM n = 74 cell bodies (for control), n = 89 cell bodies (for Atg1 OE), and n = 37 cell bodies (for Atg5 RNAi), obtained from 6 larval VNCs per genotype) **** represents p value <0.0001. C) Representative images of live larval motoneurons which co-express UAS-mCherry-GFP-Atg8A along with indicated RNAi, via the D42-Gal4 driver, in drp1 mutants (drp12/drp1KG). White dashed lines indicate the outlines of individual cell bodies. Scale bar = 10μm. D) Quantification of the sum pixel intensity of the mCherry-only signal per neuronal cell body (normalized to Control RNAi in drp1 mutant). Each point represents a single neuronal cell body, bars represent the mean ± SEM n = 76 cell bodies (for control), and n = 72 cell bodies (for Atg5 RNAi), obtained from 7 larval VNCs per genotype. **** represents p value <0.0001. (TIF) [file pgen.1009731.s008.tif]

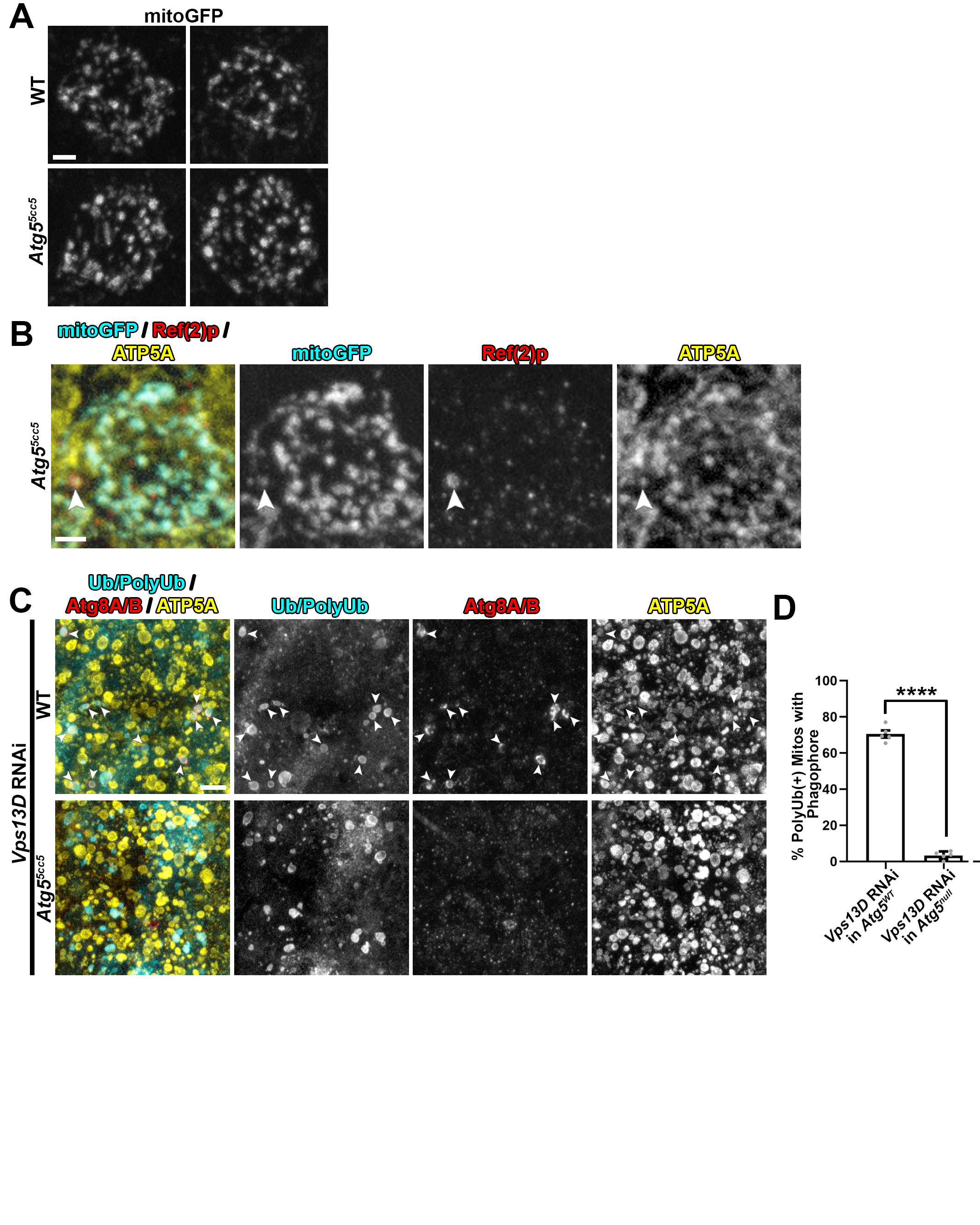

Supplement: S9 Fig — A,B) Representative images of individual dorsal midline motoneuron cell bodies which express mitoGFP (greyscale in (A), cyan in (B)) driven by the pan-motoneuron driver D42-Gal4 in WT (w1118) (top) and Atg5 null (Atg55cc5) animals (bottom). Scale bar = 2μm. B) Tissue was stained for the autophagy receptor Ref(2)p (red) and the mitochondrial IMM protein ATP5A (yellow). Arrowhead highlights a mitophagy intermediate which contains both ATP5A and mitoGFP. Scale bar = 2μm. C) Representative images of dorsal midline motoneurons which express Vps13D RNAi driven by pan-neuronal driver Elav-Gal4 in a WT (w1118) background (top panel) vs. an Atg5 null (Atg55cc5) background (bottom panel). Tissue was stained for ubiquitin (Ub/PolyUb, FK2) (cyan), phagophore protein Atg8A/B (red) and mitochondrial protein ATP5A (yellow). Arrowheads highlight polyubiquitinated mitochondria engaged with a phagophore (top panel). Scale bar = 5μm. D) Quantification of the % of polyubiquitinated mitochondria that are engaged with a phagophore. Each point represents the total percentage in the VNC from one animal, and bars represent mean ± SEM. (n = 5 for each condition, each containing >50 polyubiquitinated mitochondria). **** indicates p<0.0001. (TIF) [file pgen.1009731.s009.tif]

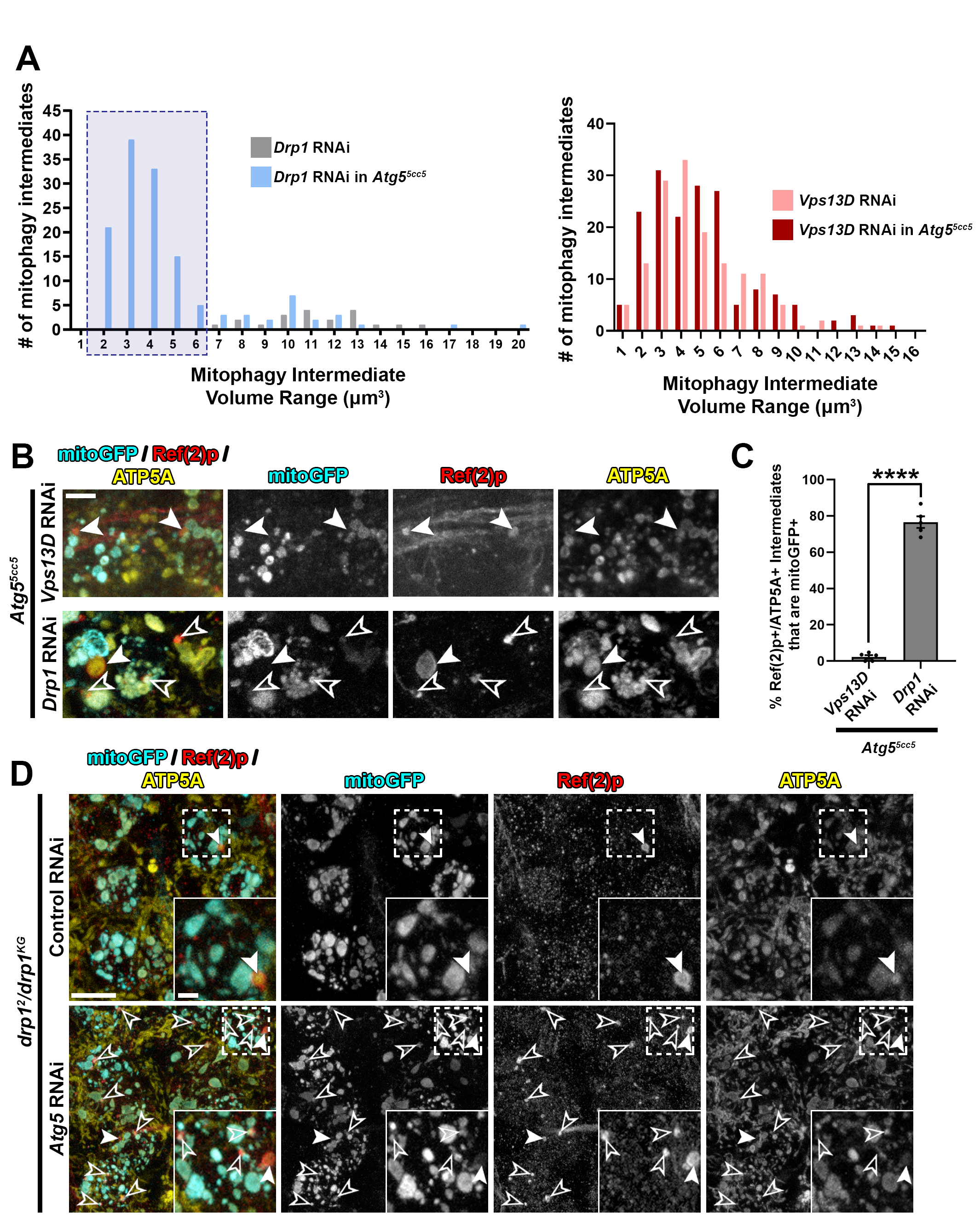

Supplement: S10 Fig — A) Histograms depicting the distribution of the volume (μm3) of mitophagy intermediates (Ref(2)p+ mitochondria) in fission-deficient conditions in WT and Atg5 mutant backgrounds. Top histogram represents conditions of Drp1 RNAi expression (n = 20 mitophagy intermediates for Drp1 RNAi condition (grey bars), and n = 136 mitophagy intermediates for Drp1 RNAi in Atg5 mutant condition (light blue bars)). Bottom histogram represents Vps13D RNAi expression (n = 168 mitophagy intermediates for Vps13D RNAi condition (pink bars) and n = 144 mitophagy intermediates in Vps13D RNAi condition in Atg5 mutants (red bars)). Blue shaded box with dashed lines indicates a population of smaller mitophagy intermediates that are revealed in Drp1 depleted neurons only when Atg5 is lost. In contrast to Drp1 RNAi neurons, no analogous new population of mitophagy intermediates was revealed when Vps13D was depleted in Atg5 mutant conditions. B) Representative images of dorsal midline motoneurons from the Atg5 mutants (Atg55cc5/y) which co-express mitoGFP (cyan) and the indicated RNAi driven by the pan-neuron driver elav-Gal4. Closed arrowheads highlight stalled mitophagy intermediates that lack mitoGFP; open arrowheads highlight stalled mitophagy intermediates that contain mitoGFP. Scale bar = 5μm. C) Quantification of the % of stalled mitophagy intermediates that contain mitoGFP. Verified Ref(2)p+/ATP5A+ objects were designated as mitoGFP+, as described in Materials and Methods in and in the legend for Fig 1C. Points represent the % of mitoGFP+ mitophagy intermediates out of total Ref(2)p+/ATP5A+ mitophagy intermediates in one animal, with n = 5 animals per RNAi condition (each condition contained >160 Ref(2)p+/ATP5A+ mitophagy intermediates). Bars represent mean ± SEM. **** indicates p<0.0001. D) Representative images of dorsal midline motoneurons from the drp1 mutants (drp12/drp1KG) which co-express mitoGFP (cyan) and the indicated RNAi driven by the pan-motoneuron driver D42-Gal4. Tissue w [file pgen.1009731.s010.tif]
